# Comparative Untargeted Metabolomic Profiling of Induced Mitochondrial Fusion in Pancreatic Cancer

**DOI:** 10.3390/metabo11090627

**Published:** 2021-09-15

**Authors:** Nicholas D. Nguyen, Meifang Yu, Vinit Y. Reddy, Ariana C. Acevedo-Diaz, Enzo C. Mesarick, Joseph Abi Jaoude, Min Yuan, John M. Asara, Cullen M. Taniguchi

**Affiliations:** 1 Department of Experimental Radiation Oncology, The University of Texas at MD Anderson Cancer Center, Houston, TX 77030, USA; nic.dk.nguyen@gmail.com (N.D.N.); yumeifang1025@gmail.com (M.Y.); vinitreddy0@gmail.com (V.Y.R.); ari.aceve12@gmail.com (A.C.A.-D.); ecm7@rice.edu (E.C.M.); JBabi@mdanderson.org (J.A.J.); 2 Department of Radiation Oncology, The University of Texas at MD Anderson Cancer Center, Houston, TX 77030, USA; 3 Division of Signal Transduction, Beth Israel Deaconess Medical Center, Boston, MA 02215, USA; myuan@bidmc.harvard.edu (M.Y.); jasara@bidmc.harvard.edu (J.M.A.); 4 Department of Medicine, Harvard Medical School, Boston, MA 02115, USA

**Keywords:** mitochondrial morphology, fusion, fission, mitofusin-2, leflunomide, pancreatic cancer, metabolomic reprogramming, metabolomics

## Abstract

Mitochondria are dynamic organelles that constantly alter their shape through the recruitment of specialized proteins, like mitofusin-2 (Mfn2) and dynamin-related protein 1 (Drp1). Mfn2 induces the fusion of nearby mitochondria, while Drp1 mediates mitochondrial fission. We previously found that the genetic or pharmacological activation of mitochondrial fusion was tumor suppressive against pancreatic ductal adenocarcinoma (PDAC) in several model systems. The mechanisms of how these different inducers of mitochondrial fusion reduce pancreatic cancer growth are still unknown. Here, we characterized and compared the metabolic reprogramming of these three independent methods of inducing mitochondrial fusion in KPC cells: overexpression of Mfn2, genetic editing of Drp1, or treatment with leflunomide. We identified significantly altered metabolites via robust, orthogonal statistical analyses and found that mitochondrial fusion consistently produces alterations in the metabolism of amino acids. Our unbiased methodology revealed that metabolic perturbations were similar across all these methods of inducing mitochondrial fusion, proposing a common pathway for metabolic targeting with other drugs.

## 1. Introduction

Pancreatic ductal adenocarcinoma (PDAC) relies on mitochondrial respiration through remodeling of the electron transport chain in order to sustain its proliferative abilities [1,2]. We and others have found that morphological changes in mitochondria can alter their function [3,4]. Mitochondria undergo fusion and fission in response to external stimuli to optimize metabolic functions and to promote turnover of damaged organelles through mitophagy [5]. This balance between mitochondrial fusion and fission is regulated by two key molecules: mitofusin-2 (Mfn2) and dynamin-related protein 1 (Drp1). As its name suggests, Mfn2 directs the fusion of outer membranes in adjacent mitochondria whereas Drp1 aggregates to the surface of elongated networks, constricting the mitochondrial membranes until they break apart through the process of mitochondrial fission.

Pancreatic cancer cells often display aberrations of mitochondrial dynamics in favor of mitochondrial fission [3,6], where these organelles take on a fragmented appearance, which appears to be a KRAS-dependent phenomenon [7]. We previously demonstrated that this overactive mitochondrial fission could be therapeutically targeted by disrupting Drp1 or increasing expression of Mfn2 genetically [3]. Leflunomide, an FDA-approved drug for rheumatoid arthritis, was identified as a pharmacologic activator of mitochondrial fusion through upregulation of Mfn2 [8]. Capitalizing on this mode of action, we also treated pancreatic cancer cells with leflunomide, which phenocopied the tumor suppressive effects of Drp1 inhibition [3]. The net effect of these three interventions (Drp1 inhibition, Mfn2 overexpression, or leflunomide) promoted mitochondrial fusion, which curbed oxidative phosphorylation (OXPHOS), thereby suppressing tumor growth in pancreatic cancer [3]. Notably, leflunomide has recently been shown to synergize with the current standard of care, gemcitabine, suggesting it as a strong candidate for potential therapeutic repurposing while harnessing the antitumor effects of mitochondrial fusion [9,10]. However, the metabolic mechanisms by which mitochondrial fusion reduce PDAC growth are unknown.

To understand how mitochondrial fusion alters the cellular metabolism of PDAC, we performed an unbiased comparative metabolomic analysis between three different methods of inducing mitochondrial fusion: (1) Genetically inducing fusion using a tetracycline-inducible system to overexpress Mfn2 (Tet-On Mfn2), (2) directly inhibiting the decomposition of mitochondrial fusion through genetically knocking out Drp1 using CRISPR (sgDrp1), and (3) pharmacologically inducing fusion through treatment with leflunomide. We found common metabolic pathways between these different methods of inducing mitochondrial fusion, suggesting areas for metabolic intervention to further optimize this therapeutic target.

## 2. Results

### 2.1. Genetic and Pharmacologic Induction of Mitochondrial Fusion

**Figure 1:**
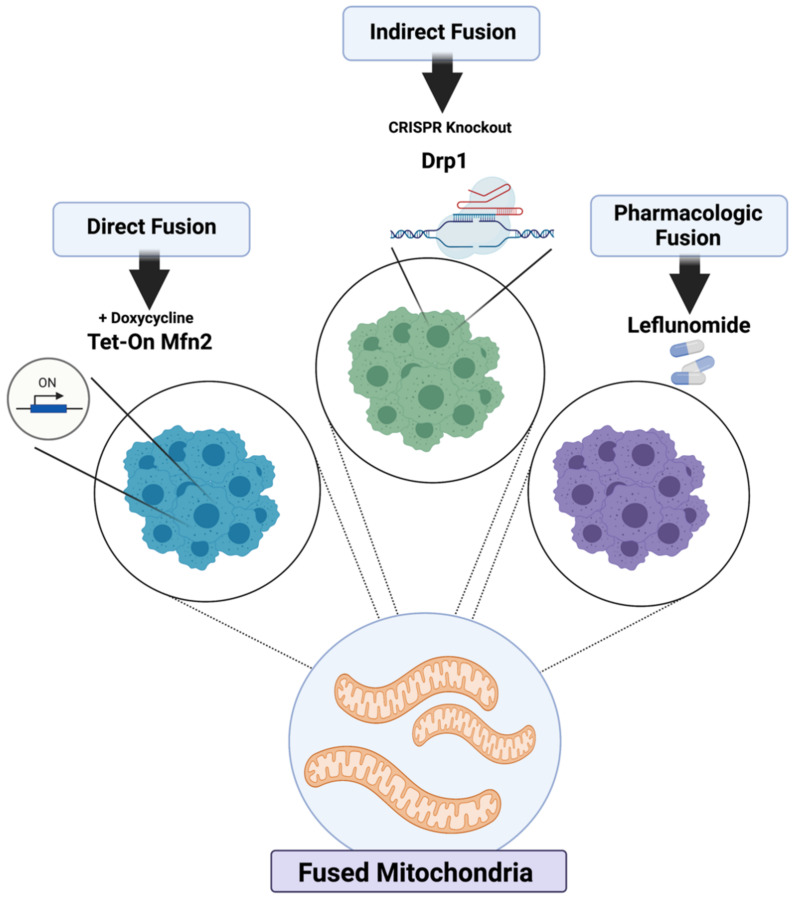
Models of mitochondrial fusion induction. KPC cells were genetically modified to directly overexpress Mfn2 in a tetracycline-inducible manner, indirectly fuse through CIRSPR knockout of Drp1, and pharmacologically fuse after treatment with Leflunomide.

We created isogenic cell lines derived from murine KPC pancreatic tumors with proven mitochondrial fusion [11]. Tet-On Mfn2 cells express Mfn2 upon exposure to low doses of doxycycline, which induces mitochondrial fusion compared to doxy-negative controls. We also produced cells with predominantly fused mitochondria by CRISPR-mediated abrogation of Drp1 or treatment with leflunomide (Figure 1).

**Figure 2:**
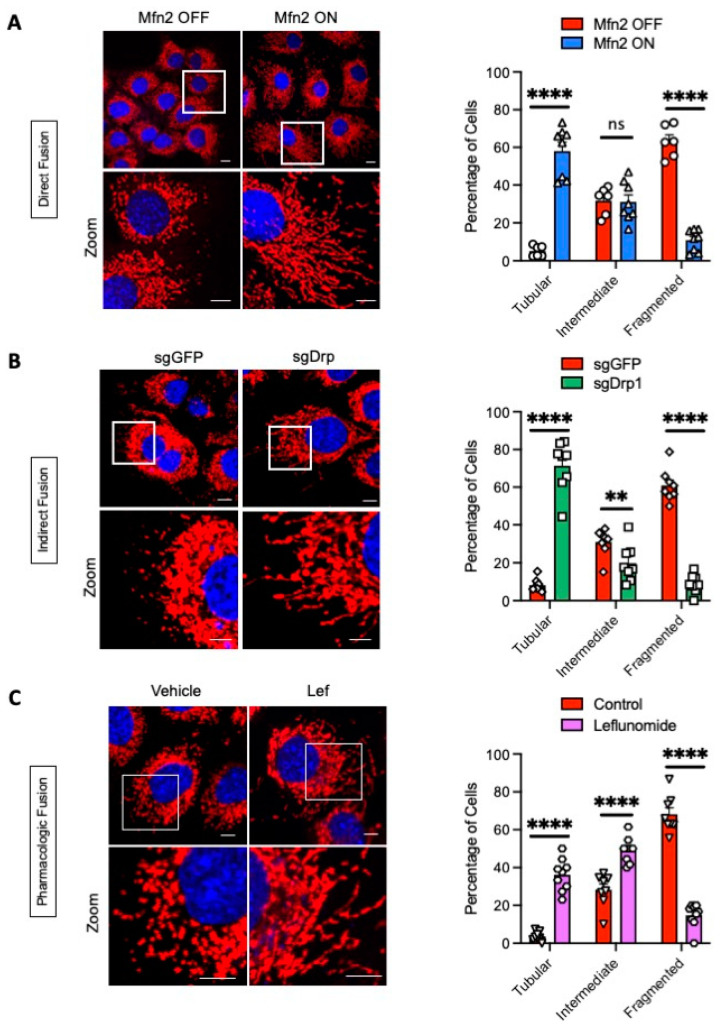
Confocal microscopy reveals changes in mitochondrial morphology in KPC cells after multiple independent fusion induction modalities. (**A**) Doxycycline-induced overexpression of Mfn2 (blue triangles) significantly increased tubular morphology while significantly decreasing fragmented mitochondria when compared to its Tet-Off control (red circles). (**B**) Indirect fusion through abrogation of Drp1 (green squares) significantly increased tubular morphology while significantly decreasing intermediate and fragmented mitochondria when compared to sgGFP controls (red diamonds). (**C**) Pharmacologic induction of fusion with Lef (purple hexagons) significantly increased tubular and intermediate mitochondrial morphology while decreasing fragmented morphology when compared to vehicle controls (red triangles). Red fluorescence represents mitochondria; blue fluorescence represents DAPI-labeled nuclei. Mitochondrial morphology quantified using *n* = 100–200 cells. Statistical analysis by Student’s *t*-test, **** *p* < 0.0001, ** *p* < 0.01. Original magnification, ×60. Scale bar = 10 µm. Data presented as mean ± SEM.

We stained live KPC cells with MitoTracker Red and quantified the morphologic changes observed under confocal microscopy (Figure 2). Tet-On Mfn2 cells exhibited a shift towards elongated mitochondria after treatment with doxycycline (Tubular = 58.1%, Fragmented = 10.9%) while the Tet-Off Mfn2 control group retained highly fragmented mitochondria (Figure 2A, Tubular = 5.2%, Fragmented = 63.1%, *p* < 0.0001). Loss of Drp1 through CRISPR gene editing maintained tubular mitochondria as opposed to its GFP knockout control (Figure 2B, sgDrp1 vs. sgGFP, Tubular: 71.4% vs. 8.1%). We observed a decrease in both intermediate and fragmented morphology after inhibition of Drp1 when compared to sgGFP (Figure 2B, sgDrp1 vs. sgGFP, Intermediate: 19.8% vs. 31%, *p* < 0.01; Fragmented: 8.8% vs. 61%, *p* < 0.0001). Treatment with leflunomide increased both tubular and intermediate mitochondrial morphology from 3.6% and 28.2%, respectively, in KPC vehicle controls to 36.1% and 49.1%, respectively (Figure 2C, *p* < 0.0001). Fragmented mitochondrial morphology decreased from 68.2% to 14.8% after treatment with leflunomide when compared to the vehicle control (Figure 2C, *p* < 0.0001).

Protein expression confirming Mfn2 overexpression in the direct fusion Tet-On Mfn2 and pharmacologic fusion leflunomide groups and loss of Drp1 in the indirect fusion sgDrp1 group is shown in Figure S1. Interestingly, Drp1 expression was not altered after selective upregulation of Mfn2 in either the Tet-On Mfn2 or leflunomide-treated groups (Figure S1A,C). In a similar fashion, Drp1 expression did not correlate with changes in Mfn2 expression (Figure S1B), suggesting that Mfn2 and Drp1 expression act independently to regulate mitochondrial morphology.

### 2.2. Mitochondrial Fusion Distinctly Alters PDAC Metabolome

We extracted metabolites from each cell line in a minimum of five biological replicates along with isogenic controls and subjected them to mass spectrometric analysis after steady-state metabolite collection using a well-established methanol extraction method [12,13].

**Figure 3:**
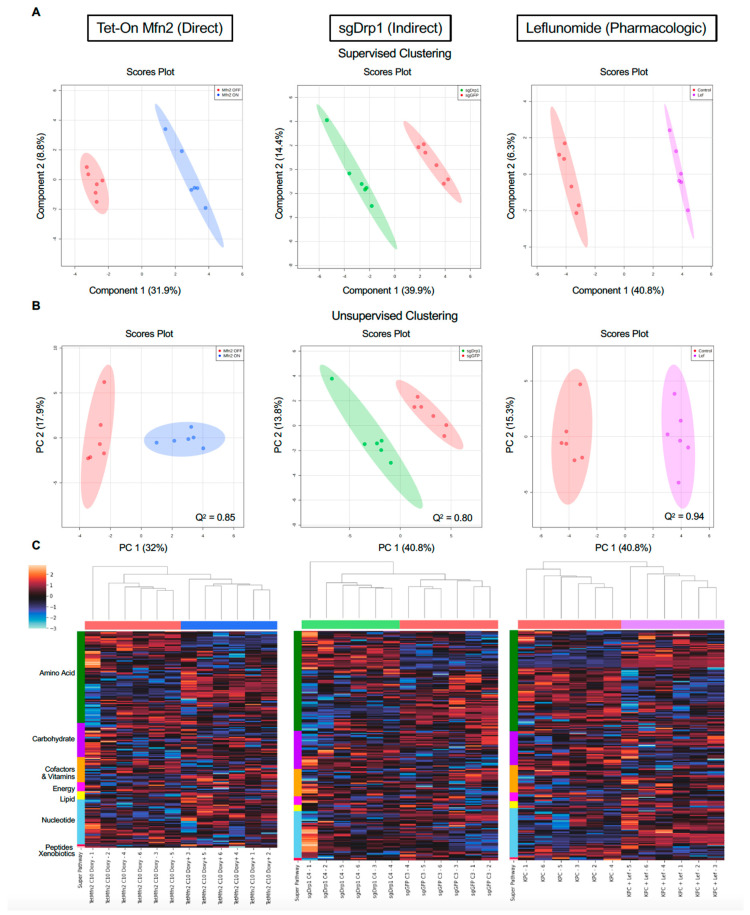
Multivariate clustering reveals distinct separation after inducing mitochondrial fusion when compared to controls. (**A**) Supervised PLS-DA and (**B**) unsupervised PCA score plots of Tet-On Mfn2 (blue), sgDrp1 (green), and Leflunomide (purple) treated KPC cells with respect to their corresponding controls (red). (**C**) Heatmap with unsupervised hierarchical clustering of affected super pathways across Tet-On Mfn2 (blue), sgDrp1 (green), and Leflunomide (purple). Both unsupervised and supervised clustering methods revealed a distinct separation between each method of fusion induction and its respective control. Predictive power of PLS-DA in component 1 represented by Q^2^ = 0.85 for Tet-On Mfn2, Q^2^ = 0.80 for sgDrp1, and Q^2^ = 0.94 for Leflunomide.

Liquid chromatography tandem mass spectrometry (LC-MS/MS) analysis quantified the relative concentration levels of 296 distinct metabolites for each of the cells. To understand the metabolites more closely correlated with mitochondrial fusion, we subjected the data to stringent filters and normalized the datasets to the control of each experimental group. Supervised partial least-squares discriminant analysis (PLS-DA) and unsupervised principal component analysis (PCA) revealed distinct clustering between the induced mitochondrial fusion groups (*n* = 6) and their respective controls (*n* = 6, Figure 3A,B). Further hierarchical clustering using a Euclidean distance and ward clustering algorithm revealed that individual replicates for each treatment group clustered together (Figure 3C). Overall, a total of 14 significantly altered metabolism pathways were shared between the Tet-On Mfn2, sgDrp1, and pharmacologically treated Leflunomide groups (Table 1). Metabolic super-pathways regulating amino acids and nucleotides were most consistently altered by the induction of mitochondrial fusion, compared to individual controls (Figure 3C).

**Figure 4:**
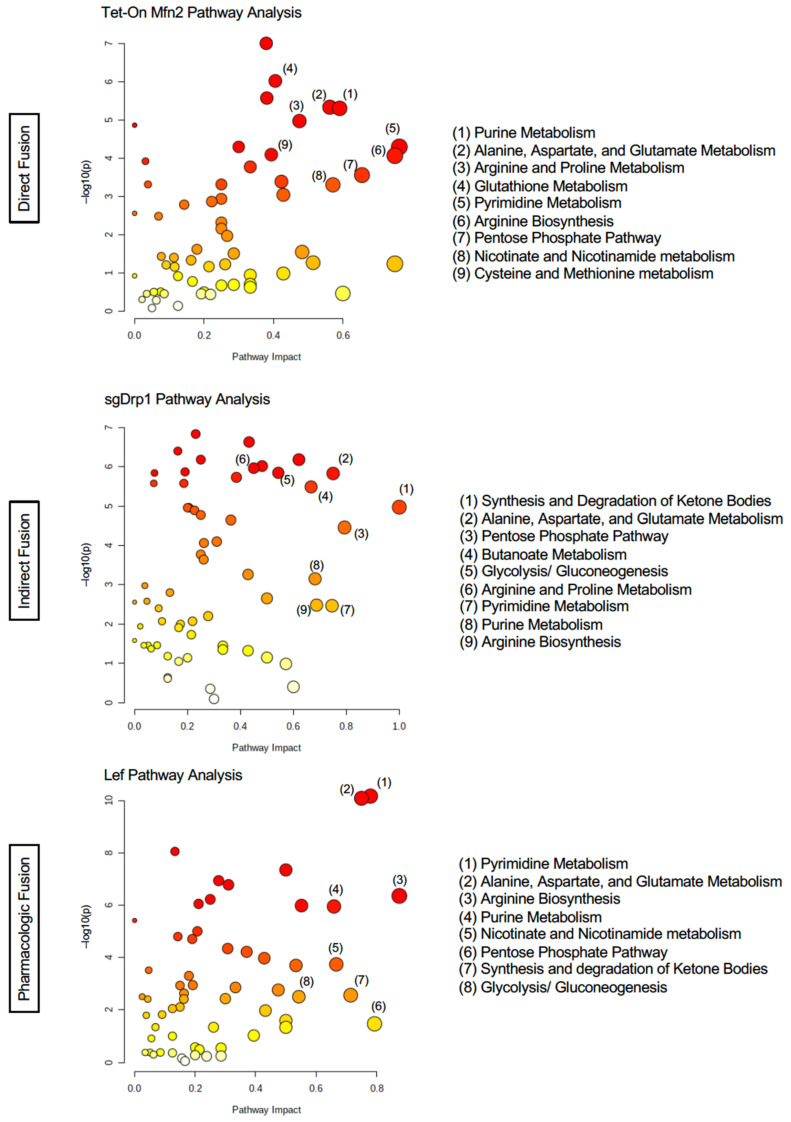
Total pathway analysis of filtered metabolites reveals similar impact of Amino Acid, Nucleotide, and Carbohydrate metabolism pathways as a function of mitochondrial fusion: Tet-On Mfn2 (direct), sgDrp1 (indirect), and leflunomide treatment (pharmacologic).

Notably, the following three amino acid pathways were significantly altered in the mitochondrial fusion cohorts compared to controls: Arginine biosynthesis (FDR < 0.01, Impact > 0.68, and Percent Affected > 78.5%), alanine, aspartate, and glutamate metabolism (FDR < 0.0001, Impact > 0.47, and Percent Affected > 57.1%), and glutathione metabolism (FDR < 0.05, Impact > 0.26, and Percent Affected > 32.1%, Table 1). Pyrimidine and purine metabolism were the two nucleotide sub-pathways that were significantly altered after inducing mitochondrial fusion (FDR < 0.01, Impact > 0.43, Percent Affected = 64.1% and FDR < 0.01, Impact > 0.48, Percent Affected > 43.9%, respectively, Table 1). We also observed significant changes in several carbohydrate metabolism sub-pathways, including the pentose phosphate pathway (PPP), glycolysis/gluconeogenesis, the citrate cycle (TCA cycle), pyruvate metabolism, and amino sugar and nucleotide sugar metabolism (Table 1). The full unfiltered pathway analysis for Tet-On Mfn2, sgDrp1, and Leflunomide-treated KPC cells can be found in Figure 4 and Table S1.

### 2.3. Identification of Significantly Differentiated Metabolites

**Figure 5:**
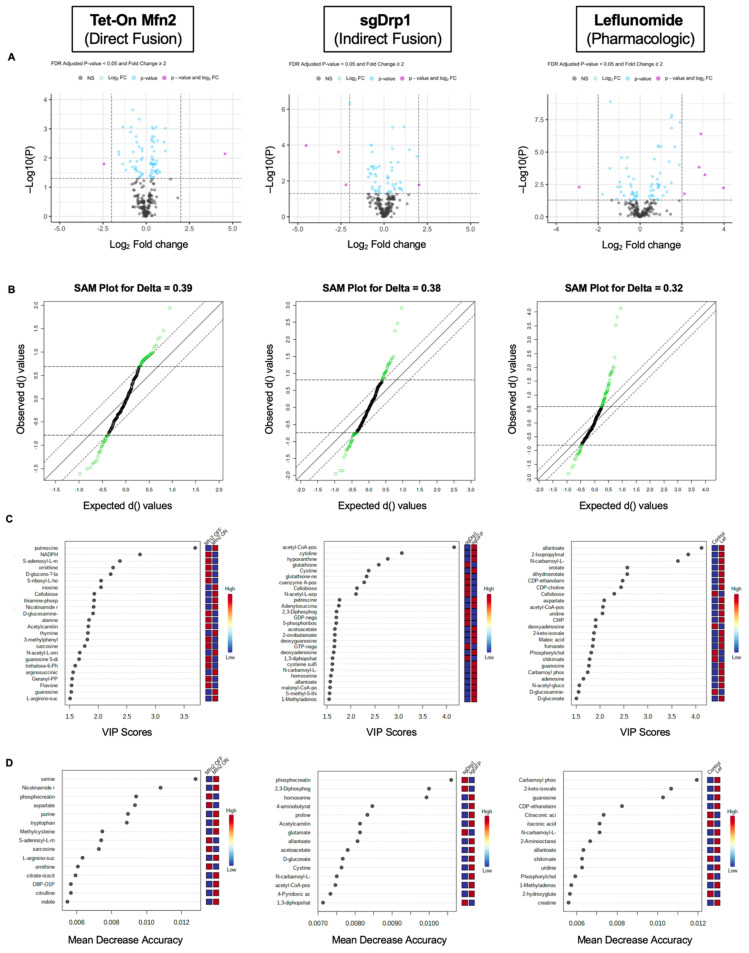
Statistical methods to identify differentially expressed metabolites after inducing mitochondrial fusion. (**A**) univariate Student’s *t*-test, FDR-adjusted *p*-value < 0.05, (**B**) SAM, FDR < 0.05, (**C**) PLS-DA, VIP score < 1.0, (**D**) RF model, Mean Decrease Accuracy > 0.

From this, we found that 75 out of 234 metabolites in Tet-On Mfn2, 54 out of 245 metabolites in the sgDrp1, and 74 out of 233 metabolites in the leflunomide-treated groups were altered (both up-and-downregulated) compared to controls. As represented in their corresponding volcano plots, since LC-MS/MS was unable to detect many metabolites with a fold change greater than 2, we repeated our analysis with a lower stringency threshold, considering all significant metabolites based on an FDR < 0.05 in our initial univariate analysis (Figure 5A). A full list of discriminant metabolites identified via Student’s *t*-test can be found in Table S2A–C.

In order to ensure robustness in our feature selection process, we further developed three different pairwise models to identify metabolite markers for each group: A significance analysis of microarray (SAM) [14], PLS-DA variable importance in projection (VIP) [15], and a random forest (RF) [16] classification model. SAM identified 73 out of 234 metabolites in the Tet-On Mfn2 induced fusion group, 71 out of 245 metabolites in the indirect fusion group, and 74 out of 233 metabolites in the leflunomide-treated group as significantly altered based on an FDR < 0.05 and a corresponding delta of 0.39, 0.38, and 0.32 for the Tet-On Mfn2, sgDrp1, and Leflunomide groups, respectively (Figure 5B). The full list of discriminant metabolites identified by SAM can be found in Table S3A–C.

Using our PLS-DA model, a VIP score greater than 1.0 [14] across all five principal components was used as a cutoff to identify discriminant metabolites after induction of mitochondrial fusion. From our original filtered metabolite set, we detected 83, 72, and 59 potential metabolites of interest in our Tet-On Mfn2, sgDrp1, and Leflunomide-treated groups accordingly (Figure 5C and Table S4A–C). Moreover, permutation testing of 2000 repeats yielded a *p*-value = 0.001, suggesting that the separation exhibited by our PLS-DA model was not due to overfitting. We then performed leave-one-out cross validation [17] of the models and found that they had a predictive power of 85% for Tet-On Mfn2, 80% for sgDrp1, and 94% for Leflunomide-induced fusion.

To account for potential overfitting and potential bias from our previous models, we also developed an RF classification model for each group using MetaboAnalyst 5.0. For each RF model, we generated 500 trees to control for potential correlations between metabolites and subsequently measured a variable permutation importance score for each metabolite represented as the mean decrease accuracy (MDA) value. An MDA value approximates the amount that our model decreases in accuracy if the variable was taken out of the model [16]. Accordingly, we classified metabolites with MDA > 0 as discriminant and included them for pathway analysis. From our models, we identified 87 total discriminant metabolites in both the Tet-On Mfn2 and sgDrp1 groups and 81 total discriminant metabolites in the Leflunomide-treated group (Figure 5D and Table S5A–C).

From our four statistical models, we combined the lists of significantly altered metabolites that contributed to each respective condition of induced mitochondrial fusion and only considered the overlap between all four lists for a definitive pathway analysis. This improved the robustness of our data analysis and further increased confidence in the identified metabolite markers for mitochondrial fusion in PDAC. As a result, we uncovered 48 unique identifier metabolites for direct fusion by Tet-On Mfn2 (Table 2), 38 unique identifier metabolites for indirect fusion by CRISPR knockout of Drp1 (Table 3), and 47 unique identifier metabolites for pharmacologic fusion by Leflunomide (Table 4).

### 2.4. Targeted Pathway Analysis Distinguishes Altered Metabolome after Mitochondrial Fusion

**Figure 6:**
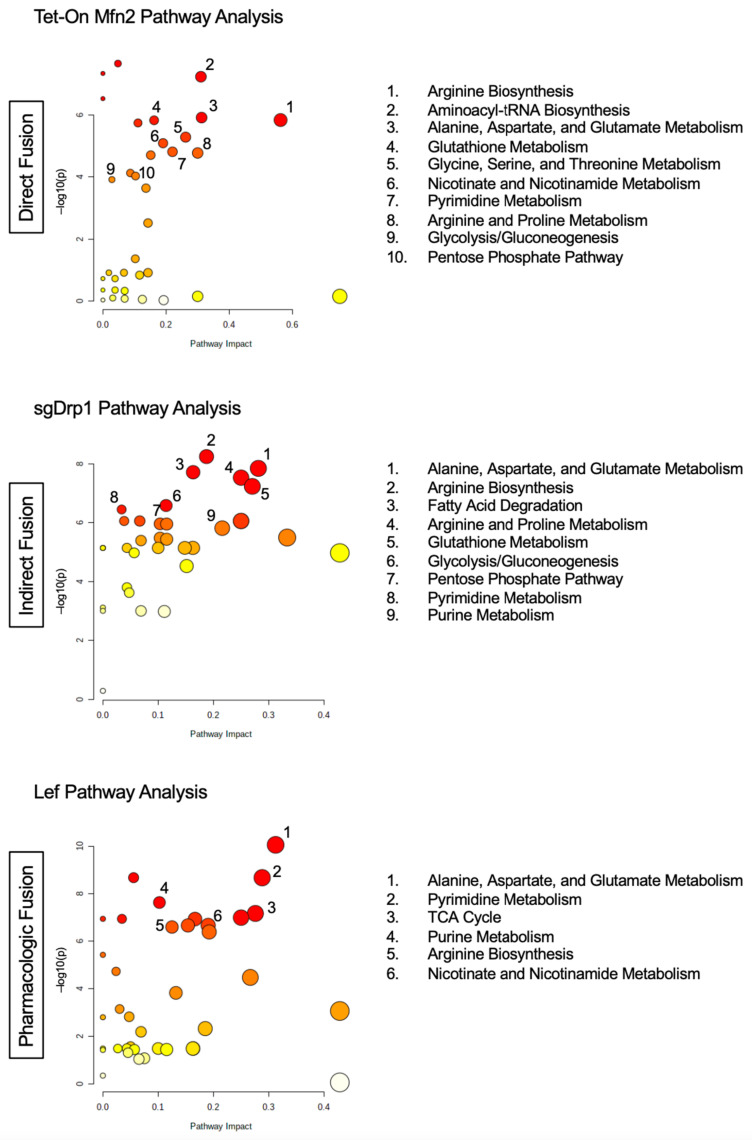
Pathway analysis of overlapped discriminant metabolites from direct fusion in Tet-On Mfn2, indirect fusion in sgDrp1, and pharmacologic fusion in leflunomide-treated KPC cells.

When conducting sub-pathway analysis from each list of discriminant metabolites identified via one of our four statistical models, we found that the overarching patterns observed in alterations of Amino Acid, Nucleotide, and Carbohydrate super-pathways remained similar to those of our initial untargeted sub-pathway analysis. More importantly, sub-pathway analysis from each distinct discriminant metabolite identification method appeared to yield very similar phenotypes across direct genetic fusion, indirect genetic fusion, and pharmacologic fusion (Figures S2–S5 and Tables S6–S9(A–C)). Using our overlapped discriminant metabolite list for sub-pathway analysis, we discovered that even our more limited metabolite set was able to recapitulate these trends in metabolic reprogramming in each independent method of mitochondrial fusion induction. Specifically, Amino Acid, Nucleotide, and Carbohydrate pathways were altered across all three experimental groups (Figure 6). We found eight particular sub-pathways that were considered significantly impacted after filtering the raw pathway outputs from MetaboAnalyst based on an FDR < 0.05 (Table S10A–C). These included alanine, aspartate, and glutamate metabolism (FDR < 0.0001), arginine biosynthesis (FDR < 0.0001), glutathione metabolism (FDR < 0.05), cysteine and methionine metabolism (FDR < 0.01), pyrimidine metabolism (FDR < 0.0001), purine metabolism (FDR < 0.0001), PPP (FDR < 0.001), and glycolysis/gluconeogenesis (FDR < 0.05, Table 5).

Further analysis revealed that although these pathways were considered statistically significant based on our FDR adjusted *p*-value, we observed that many of these pathways had a low impact and correspondingly low percentage of affected metabolites in one of the three groups. Diving deeper, arginine biosynthesis appeared to be more affected after direct fusion with Tet-On Mfn2 (Impact = 0.56, Percent Affected = 42.9%) than after indirect fusion with sgDrp1 (Impact = 0.19, Percent Affected = 14.3%) and pharmacologic fusion with Leflunomide (Impact = 0.13, Percent Affected = 21.4% Table 5). Glutathione metabolism was more heavily affected by indirect fusion (Impact = 0.27, Percent Affected = 17.9%) than direct fusion (Impact = 0.16, Percent Affected = 14.3%) and pharmacologic fusion (Impact = 0.03, Percent Affected = 3.6%). Cysteine and methionine metabolism was least impacted by Leflunomide treatment (Impact = 0.03, Percent Affected = 3.0%) followed by Drp1 knockout (Impact = 0.15, Percent Affected = 12.1%) and Mfn2 upregulation (Impact = 0.15, Percent Affected = 15.2%, Table 5).

Understandably, pyrimidine metabolism was most affected by leflunomide treatment (Impact = 0.28, Percent Affected = 25.6%) since its mechanism of action directly inhibits dihydroorotate dehydrogenase (DHODH), a crucial enzyme in the de novo pyrimidine biosynthesis pathway. Interestingly, direct fusion through Tet-On Mfn2 closely mirrored Leflunomide’s effect on pyrimidine metabolism, with an impact of 0.22 on the pathway with 23.1% of its metabolites altered (Table 5). Purine metabolism was most affected by indirectly inducing fusion with knockout of Drp1 (Impact = 0.22, Percent Affected = 15.2%); though, still moderately altered via direct fusion with Tet-On Mfn2 (Impact = 0.14, Percent Affected = 10.6%) and pharmacologic fusion with leflunomide (Impact = 0.10, Percent Affected = 9.1%, Table 5). Both carbohydrate metabolism sub-pathways, PPP and glycolysis, were modestly impacted across the three groups, but knockdown of Drp1 in particular had more impact on glycolysis/gluconeogenesis (Impact = 0.11, Percent Affected = 11.5%), supporting recent findings that Drp1 promotes metabolic changes through glycolysis to drive PDAC tumorigenesis (Table 5) [7,18]. The main pathway that had an impact greater than 0.28 across the Tet-On Mfn2, sgDrp1, and Leflunomide groups was alanine, aspartate, and glutamate metabolism with more than 21.4% of the pathway appearing significantly altered (Table 5).

Interestingly, several pathways from this analysis were identified as specific to each independent method for mitochondrial fusion induction. We noticed that direct fusion via Tet-On Mfn2 showed a distinct impact on aminoacyl-tRNA biosynthesis and glycine, serine, and threonine metabolism in the top five pathway hits (Figure 6). Likewise, fatty acid degradation and the citrate cycle (TCA cycle) were specific to the sgDrp1 and Leflunomide groups when considering only the top five pathway hits (Figure 6). Nevertheless, after mapping the significantly altered metabolic pathways identified from the KEGG database, using our overlapped discriminant metabolite set exhibited that they were in fact highly interconnected (Figure S6). Alanine, aspartate, and glutamate metabolism fed into each of the previously mentioned metabolic pathways, aligning with each of our metabolic screens. Furthermore, we see that many of the pathways are interdependent among each other, suggesting that altering mitochondrial morphology from a punctate to fused state does in fact play a significant role in metabolic reprogramming in favor of curbing tumorigenesis.

## 3. Discussion

Advances in mitochondrial biology in the previous decade have opened the doors to novel means of therapeutically targeting tumorigenesis. It has been widely shown that mitochondrial respiration is essential across multiple tumor-types in order to circumvent limitations in glycolysis, actively remodeling their means for cellular energetics [2,19,20,21]. This is particularly true in pancreatic cancer where mitochondrial dysfunction has been found to shift the cellular bioenergetics of cells to favor OXPHOS, supporting proliferation and metastasis [22,23]. Moreover, we and others have shown that defects in *KRAS*, the most widely mutated gene in PDAC, is characteristic of fragmented mitochondria [3,7,23]. Although more than 90% of PDAC cases are driven by oncogenic *KRAS* [24], limited advancements have been made in formulating a clinical approach to target the gene. Instead, our work provided an alternative solution, altering the phenotypic state of mitochondria driven by KRAS through Drp1, which in turn suppresses tumor growth through modulating levels of defective mitochondria and limiting OXPHOS capability in PDAC [3]. In order to gain a better understanding of the impact of shifting the status of mitochondria from fragmented to fused, we attempted to elucidate the metabolome of PDAC after three independent methods of inducing fusion. To our knowledge, this is the first comparative metabolomic study of mitochondrial fusion in pancreatic cancer to identify macroscopic pathway alterations.

Our study builds upon previous findings to better understand common metabolic perturbations from mitochondrial fusion. Here, we used three different models: A direct fusion model involving the upregulation of Mfn2 in a doxycycline-dependent manner, an indirect fusion model through the attenuation of Drp1 using CRISPR-Cas9, and a pharmacologic approach with leflunomide, each in KPC cells. Of note, alanine, aspartate, and glutamate metabolism was the most prominent hit across all three methods of induced fusion, understandably because of its overarching position providing the precursors for the other seven identified metabolic pathways commonly altered (Figure 6 and Figure S5). This finding further supports current working theories of tumor ability to alternatively fuel metabolism using extracellular amino acid pools, particularly, alanine, aspartate, glutamate, and asparagine, as carbon sources [13,25]. The synthesis of these non-essential amino acids drive the formation of key oncogenic metabolites through activity of the TCA cycle [26] or altering the redox state of the cell [27]. Aspartate-derived asparagine via asparagine synthetase is also one of the two primary methods for PDAC cells to receive asparagine, without which leads these cells to undergo apoptosis [28,29].

We also observed several expected alterations in nucleotide pathways downstream due to dysregulation of glycolysis and PPP that are linked to mutant KRAS activation [23,30,31,32] by reducing the pool of fragmented mitochondria present. Leflunomide is widely known for its direct inhibition of DHODH, an enzyme localized on the inner mitochondrial membrane that is responsible for de novo pyrimidine biosynthesis. Interestingly, genetic modulation of mitochondrial morphology appeared to affect these pathways in a similar fashion. Tet-On Mfn2 and sgDrp1 modulated pyrimidine biosynthesis in addition to glycolysis and PPP, showing a common set of metabolic disturbances across multiple super-pathways. Ultimately, this suggests that mitochondrial fusion may work in tandem with DHODH inhibition as a tumor suppressive mechanism in pancreatic cancer.

Nevertheless, future research is still needed in order to fully characterize the mechanism by which mitochondrial fusion reprograms the metabolome to curb tumorigenesis. We recognize that our study follows a markedly stringent method of discriminant metabolite identification. Relative metabolite concentration readings missed at least one data point for more than 20% of our metabolites across all three induced fusion groups as a result of either low concentration or poor mass spectrometry signal response, potentially limiting our analysis. Methods to impute these missing values should be explored when doing an in-depth analysis of sub-pathway alterations. However, our study provides foundational evidence that mitochondrial morphology plays a notable role in metabolic reprogramming, further supporting leflunomide as a novel therapeutic against PDAC due to its ability to leverage both mitochondrial fusion and DHODH inhibition.

## 4. Materials and Methods

### 4.1. Cell Culture

Murine KPC cells syngeneic with C57BL/6 (K8484) were a generous gift from Anirban Maitra from the University of Texas MD Anderson Cancer Center. KPC cells were grown in RPMI-1640 supplemented with 10% FBS, 2 mM GlutaMax, 1 mM sodium pyruvate, and 7 µg/mL of insulin. We previously described the generation and selection of KPC Tet-On Mfn2, sgDrp1, and sgGFP clones [3].

### 4.2. Confocal Microscopy and Mitochondrial Morphology Analysis

KPC cells were prepared for confocal microscopy using 25 nM of MitoTracker Red CMXRos and mounted in mounting medium containing DAPI as previously described [3]. Cells were visualized using an Olympus FV1000 confocal microscope, processed using Olympus’s Fluoview software (Center Valley, PA, USA), and mitochondrial morphology was scored with *n* = 100–200 cells per group. Morphology was characterized into three different categories: Tubular, fragmented, and intermediate. Tubular scoring consisted of cells with greater than 80% elongated mitochondria. Intermediate scoring consisted of cells with greater than 50% short-rod like mitochondria, and fragmented scoring consisted of cells with greater than 50% punctate mitochondria [3].

### 4.3. Immunoblot Analysis

Tetracycline-inducible Mfn2 and Drp1 knockout tumors were lysed using T-PER Tissue Protein Extraction Reagent from Thermo Fisher Scientific (Waltham, MA, USA). Similarly, KPC cell lines treated with leflunomide were lysed using M-PER Mammalian Protein Extraction Reagent from Thermo Fisher Scientific. Lysates were run on Any kD mini-protean TGX Pre-cast protein gels from Bio-Rad (Hercules, CA, USA) and transferred onto PVDF with a Bio-Rad Trans-Blot Turbo transfer system as previously described [3,11]. Blots to probe for Mfn2 and Drp1 were run concurrently in the same apparatus in order to simultaneously blot for both proteins. Vinculin was used as the loading control. Primary antibodies against Vinculin, Mfn2, and Drp1 were purchased from Cell Signaling Technology (Beverly, MA, USA) [3]. HRP-conjugated secondary antibodies were from Thermo Fisher Scientific to probe for primary antibodies. We used the Pierce™ ECL Western Blotting Substrate from Thermo Fisher Scientific (Waltham, MA, USA) for chemiluminescence detection on a ChemiDoc imaging system by Bio-Rad (Hercules, CA, USA).

### 4.4. Untargeted Metabolomic Analysis

All metabolomic analyses were conducted under steady-state conditions. KPC cell lines were grown in appropriate growth media in six replicates in 10-cm plates. Two hours before metabolite collection, cells were incubated in fresh growth media. Accordingly, replicate cell lines were plated and grown in parallel in order to control for cell growth. Cell counts from the replicate plates were used to normalize metabolite readings. After incubation in fresh growth media, 4 mL of 80% methanol that was pre-chilled to −80 °C was added, and the cell plates were immediately transferred to −80 °C to incubate overnight. The cell lysate-methanol mixture was then scraped and transferred to conical tubes on dry ice and centrifuged at 5000× *g* for 5 min. The supernatant was collected, and the process was repeated two more times after resuspending the pellet in 500 µL of chilled 80% methanol, for a total volume of 5 mL. Samples were then completely dried via speed vacuum at 30 °C. Metabolites were analyzed using a 5500 QTRAP LC-MS/MS system (SCIEX, Framingham, MA, USA) via selected reaction monitoring (SRM). Mass spectrometric peak area integration was done using MultiQuant 2.0 software (SCIEX, Framingham, MA, USA) as previously described [12,13].

### 4.5. Discriminant Metabolite Identification

From our initial 296 measured metabolites, we stringently filtered out readings that were missing any values to ensure robustness in our analysis. As a result, 79.1%, 82.8%, and 78.7% of the metabolites within the datasets remained for our Tet-On Mfn2, sgDrp1, and Leflunomide groups, respectively. Continued filtering for altered metabolites was performed using four independent statistical approaches: (1) FDR-adjusted two-sided Student’s *t*-test (*p*-values < 0.05 were considered statistically significant), (2) SAM (significant metabolite features were identified at an FDR < 0.05 and corresponding delta of 0.39 for Tet-On Mfn2, 0.38 for sgDrp1, and 0.32 for Leflunomide), (3) PLS-DA variable importance in projection (VIP scores > 1.0 were considered significant for class separation), and (4) RF classification based on 500 trees (significance established at permutation importance, MDA > 0).

Two-sided Student’s *t*-test characterized significant differences based on hypothesis testing of the groups’ means following a normal distribution. However, since we only had *n* = 6 for each group, there is the possibility that the variance in our dataset is not stable [33]. To account for this, SAM uses a nonparametric approach, which does not rely on a prescribed probability distribution [34,35]. SAM processes multiple permutations of our data in order to calculate FDR values, which we are able to control using the tuning parameter delta, allowing us to define our cutoff for identification of altered metabolites [36]. PLS-DA VIP measures the importance of each variable after supervised dimensional reduction using a partial least squares projection [37,38]. Given that the average squared VIP score is 1.0, we followed the field standard of considering VIP scores greater than 1.0 as significantly altered and confirmed its predictive probabilities using a leave-one-out cross validation method [39]. RF is a machine learning model often used for regression and classification. We tuned an RF model using bootstrap sampling to generate 500 random classification trees. Since this model only uses a subset of the available data to generate trees, we were able to robustly limit overfitting as well as potential outliers [40]. An MDA score was calculated using the unbiased out-of-bag classification error for each metabolite predictor, representing its predictive importance for the model. The reference MDA of 0 signifies that the predictor has no predictive importance in the model. Therefore, a metabolite with an MDA > 0 represents that the loss of that metabolite from the model will result in a decrease in predictive ability of the RF model, which we used as our cutoff for identifying discriminant metabolites. In order to confirm our pathway analysis findings from the discriminant metabolite lists generated from each of our statistical models, we further refined our list of metabolites by taking only those that were common across all four feature selection methods.

### 4.6. Pathway Analysis

Custom data mining using BioPython’s KEGG API was used to collect super metabolic pathway data for hierarchical clustering similar to what has previously been described [41]. Further sub-pathway analysis was performed using MetaboAnalyst 5.0, initially on the total filtered metabolite set from the KEGG database for mus musculus. We used a Global Test for enrichment and out-degree centrality for topology analysis. Using the hit values from the MetaboAnalyst output, we calculated a percent affected score for each pathway to prevent identification of significantly altered pathways with fewer than 20% of their metabolites altered. Significant sub-pathways were also filtered using an FDR adjusted *p*-value < 0.05 and impact greater than 0.25. Confirmation of sub-pathway alterations after the induction of mitochondrial fusion was performed after running continued pathway analyses of each discriminant metabolite set generated from our four different statistical methods as well as our overlapped metabolite dataset.

### 4.7. Statistical Analysis

Data manipulation and statistical analyses were performed using MetaboAnalyst 5.0 [42], R version 4.0.2, and the Pandas, NumPy, and SciPy libraries in python 3.8. Metabolite concentration values were normalized based on the control group for each respective experimental group and log-transformed and pareto-scaled to approximate a normal distribution. 2D PCA and PLS-DA scores plots were generated using MetaboAnalyst. Heatmaps were generated with the python Seaborn package using a Euclidean distance measure and ward algorithm. Volcano plots were generated using the EnhancedVolcano package from Bioconductor in R [43].

## 5. Conclusions

Comparative metabolomic analysis of KPC cells exhibiting induced mitochondrial fusion after direct overexpressing Mfn2, indirect inhibition of Drp1, and pharmacologic treatment with leflunomide revealed similar alterations in the metabolome. Notably, we observed changes in several key metabolic amino acid pathways implicated in cancer progression, supporting current literature showing the oncogenic role of mitochondrial fission in pancreatic cancer, and the potential value of shifting mitochondrial morphology to a fused state. Furthermore, this avenue for potential pancreatic cancer treatment is within reach through the FDA-approved drug leflunomide, which has been established as a mitochondrial fusion activator.

## Figures and Tables

**Table 1:** Common altered metabolic pathways from the initial metabolite set across pathways supporting mitochondrial fusion. Pathway analysis included only pathways with an FDR < 0.05, impact > 0.25, and more than 20% of the metabolites in the pathway affected.

Pathway Name	Tet-On Mfn2 (Direct Fusion)				sgDrp1 (Indirect Fusion)				Leflunomide (Pharmacologic)			
	Percent Affected	Differentiated Metabolites	FDR	Impact	Percent Affected	Differentiated Metabolites	FDR	Impact	Percent Affected	Differentiated Metabolites	FDR	Impact
Pyrimidine Metabolism	64.1%	25/39	3.39 × 10^−4^	0.821	64.1%	25/39	5.95 × 10^−3^	0.746	64.1%	25/39	2.43 × 10^−9^	0.432
Arginine Biosynthesis	78.6%	11/14	4.69 × 10^−4^	0.802	85.7%	12/14	5.91 × 10^−3^	0.688	92.9%	13/14	3.73 × 10^−6^	0.750
Pentose Phosphate Pathway	59.1%	13/22	1.18 × 10^−3^	0.758	63.6%	14/22	9.65 × 10^−5^	0.793	63.6%	14/22	5.02 × 10^−2^	0.659
Alanine, Aspartate, and Glutamate Metabolism	57.1%	16/28	6.00 × 10^−5^	0.731	64.3%	18/28	8.16 × 10^−6^	0.750	64.3%	18/28	2.43 × 10^−9^	0.475
Glycolysis/Gluconeogenesis	50%	13/26	9.43 × 10^−2^	0.643	53.9%	14/26	8.16 × 10^−6^	0.543	53.9%	14/26	6.18 × 10^−3^	0.780
Synthesis and Degradation of Ketone Bodies	40%	2/5	6.16 × 10^−2^	0.600	100%	5/5	3.72 × 10^−5^	1.000	60%	3/5	5.79 × 10^−3^	0.875
Glyoxylate and Dicarboxylate Metabolism	34.4%	11/32	9.43 × 10^−2^	0.556	43.8%	14/32	9.40 × 10^−6^	0.385	34.4%	11/32	1.67 × 10^−4^	0.793
Citrate Cycle (TCA cycle)	50%	10/20	5.80 × 10^−2^	0.513	65%	13/20	7.97 × 10^−6^	0.621	60%	12/20	6.00 × 10^−6^	0.308
Purine Metabolism	43.9%	29/66	6.00 × 10^−5^	0.483	51.5%	34/66	1.53 × 10^−3^	0.682	50%	33/66	6.00 × 10^−6^	0.667
Arginine and Proline Metabolism	36.8%	14/38	1.07 × 10^−4^	0.469	39.5%	15/38	8.16 × 10^−6^	0.450	36.8%	14/38	3.94 × 10^−3^	0.552
Nicotinate and Nicotinamide Metabolism	53.3%	8/15	1.65 × 10^−3^	0.461	53.33%	8/15	1.13 × 10^−1^	0.571	60%	9/15	5.65 × 10^−4^	0.714
Amino Sugar and Nucleotide Sugar Metabolism	24.3%	9/37	9.81 × 10^−2^	0.434	24.3%	9/37	5.33 × 10^−4^	0.261	24.3%	9/37	6.36 × 10^−2^	0.543
Glutathione Metabolism	32.1%	9/28	2.89 × 10^−5^	0.346	35.7%	10/28	7.08 × 10^−6^	0.432	35.7%	10/28	1.74 × 10^−2^	0.261
Pyruvate Metabolism	31.8%	7/22	1.65 × 10^−1^	0.251	45.5%	10/22	8.16 × 10^−6^	0.481	31.8%	7/22	2.10 × 10^−4^	0.370

**Table 2:** Discriminant metabolites identified after induction of Mfn2. Test statistics calculated for significantly altered metabolites overlapped across the univariate Student’s *t*-test, SAM, PLS-DA, and RF analysis.

Sample Name	Fold Change	Univariate FDR	VIP Score (Comp 1)	Mean Decrease Accuracy (MDA)	SAM FDR
CDP	0.687	2.78 × 10^−2^	1.287	6.67 × 10^−4^	3.21 × 10^−2^
Carbamoyl Phosphate	1.251	2.61 × 10^−2^	1.008	1.80 × 10^−3^	5.04 × 10^−2^
Asparagine	1.264	1.13 × 10^−2^	1.065	3.40 × 10^−3^	3.74 × 10^−2^
D-Glucosamine-1-Phosphate	0.443	2.61 × 10^−2^	1.909	2.33 × 10^−3^	1.37 × 10^−2^
S-Adenosyl-L-Methioninamine	0.329	6.07 × 10^−3^	2.379	2.27 × 10^−3^	4.84 × 10^−3^
2-Dehydro-D-Gluconate	0.686	2.86 × 10^−3^	1.396	9.93 × 10^−3^	2.35 × 10^−2^
Indole	1.363	2.86 × 10^−3^	1.267	3.00 × 10^−3^	2.82 × 10^−2^
Citrulline	1.270	1.84 × 10^−3^	1.135	5.53 × 10^−3^	3.14 × 10^−2^
GTP	1.337	1.60 × 10^−2^	1.156	1.00 × 10^−3^	3.55 × 10^−2^
Arginosuccinic Acid	1.584	2.57 × 10^−3^	1.561	5.93 × 10^−3^	1.42 × 10^−2^
GMP	1.419	1.59 × 10^−2^	1.303	1.80 × 10^−3^	2.99 × 10^−2^
2-Aminooctanoic Acid	0.670	1.84 × 10^−3^	1.463	3.60 × 10^−3^	1.85 × 10^−2^
Arginine	1.254	2.28 × 10^−3^	1.101	3.40 × 10^−3^	3.32 × 10^−2^
Purine	1.265	4.14 × 10^−3^	1.099	5.00 × 10^−3^	3.41 × 10^−2^
NADPH	0.185	1.59 × 10^−2^	2.729	2.63 × 10^−3^	4.84 × 10^−3^
O8P-O1P	1.378	5.76 × 10^−3^	1.276	6.53 × 10^−3^	2.91 × 10^−2^
L-Arginino-Succinate	1.496	8.71 × 10^−4^	1.508	8.44 × 10^−3^	1.37 × 10^−2^
Alanine	0.548	8.71 × 10^−4^	1.839	1.48 × 10^−2^	4.84 × 10^−3^
5-Phosphoribosyl-1-Pyrophosphate	1.598	2.59 × 10^−2^	1.458	6.67 × 10^−4^	2.80 × 10^−2^
S-Ribosyl-L-Homocysteine	0.391	1.62 × 10^−2^	2.049	3.13 × 10^−3^	9.16 × 10^−3^
Acetylcarnitine DL	0.535	1.29 × 10^−3^	1.827	3.27 × 10^−3^	4.84 × 10^−3^
2-Hydroxy-2-Methylbutanedioic Acid	1.402	1.59 × 10^−2^	1.282	2.33 × 10^−3^	2.99 × 10^−2^
Glutathione Disulfide	1.314	1.98 × 10^−2^	1.097	8.93 × 10^−3^	3.74 × 10^−2^
Phenylalanine	1.283	8.91 × 10^−4^	1.172	1.23 × 10^−2^	2.92 × 10^−2^
dTMP	1.118	2.03 × 10^−2^	1.441	5.60 × 10^−3^	2.80 × 10^−2^
NADH	1.246	1.25 × 10^−2^	1.434	6.67 × 10^−4^	2.60 × 10^−2^
Nicotinamide Ribotide	2.050	2.86 × 10^−3^	1.920	2.33 × 10^−3^	4.84 × 10^−3^
Uridine	1.401	2.78 × 10^−2^	1.229	1.40 × 10^−3^	3.41 × 10^−2^
Indoleacrylic Acid	1.317	1.15 × 10^−2^	1.168	4.80 × 10^−3^	3.41 × 10^−2^
Tryptophan	1.374	1.84 × 10^−3^	1.303	6.73 × 10^−3^	2.60 × 10^−2^
3-Phosphoglycerate	0.689	1.28 × 10^−2^	1.336	5.13 × 10^−3^	2.91 × 10^−2^
N-Acetyl-Glucosamine	0.663	2.43 × 10^−2^	1.343	1.30 × 10^−3^	2.99 × 10^−2^
Sarcosine	0.582	2.23 × 10^−4^	1.763	5.80 × 10^−3^	4.84 × 10^−3^
Tyrosine	1.259	8.89 × 10^−3^	1.066	1.17 × 10^−2^	3.72 × 10^−2^
Aspartate	0.759	4.67 × 10^−4^	1.250	5.13 × 10^−3^	2.60 × 10^−2^
D-Glucono-1,5-Lactone-6-Phosphate	0.338	7.52 × 10^−3^	2.217	3.60 × 10^−3^	4.84 × 10^−3^
Methylcysteine	1.333	1.00 × 10^−3^	1.256	2.00 × 10^−3^	2.60 × 10^−2^
Glycerophosphocholine	1.321	1.61 × 10^−2^	1.138	4.27 × 10^−3^	3.55 × 10^−2^
Putrescine	23.644	7.14 × 10^−3^	3.693	3.33 × 10^−3^	0.00 × 10^0^
Ornithine	0.394	8.71 × 10^−4^	2.259	6.33 × 10^−3^	4.84 × 10^−3^
Trehalose-6-Phosphate	0.578	2.03 × 10^−2^	1.598	1.07 × 10^−3^	2.35 × 10^−2^
Carnitine	0.748	4.14 × 10^−3^	1.231	5.27 × 10^−3^	2.93 × 10^−2^
Pantothenate	1.474	1.62 × 10^−2^	1.327	3.93 × 10^−3^	2.93 × 10^−2^
Serine	1.260	2.57 × 10^−3^	1.115	4.40 × 10^−3^	3.29 × 10^−2^
Guanosine	1.721	3.04 × 10^−2^	1.530	8.00 × 10^−4^	2.60 × 10^−2^
Inosine	2.150	9.67 × 10^−4^	2.047	9.93 × 10^−3^	4.84 × 10^−3^
Orotidine-5-Phosphate	1.499	2.79 × 10^−2^	1.355	2.33 × 10^−3^	2.99 × 10^−2^
Thiamine-Phosphate	2.167	5.76 × 10^−3^	1.927	5.80 × 10^−3^	7.20 × 10^−3^

**Table 3:** Discriminant metabolites identified after deletion of Drp1. Test statistics calculated for significantly altered metabolites overlapped across the univariate Student’s *t*-test, SAM, PLS-DA, and RF analysis.

Sample Name	Fold Change	Univariate FDR	VIP Score (Comp 1)	Mean Decrease Accuracy (MDA)	SAM FDR
Betaine	0.664	2.15 × 10^−3^	1.413	8.60 × 10^−3^	7.70 × 10^−3^
4-Pyridoxic Acid	0.725	3.76 × 10^−2^	1.130	1.33 × 10^−3^	3.30 × 10^−2^
Phosphocreatine	1.317	9.06 × 10^−4^	1.174	5.27 × 10^−3^	1.41 × 10^−2^
Aminoimidazole Carboxamide Ribonucleotide	0.630	2.83 × 10^−2^	1.403	1.67 × 10^−3^	1.41 × 10^−2^
Glutathione	3.743	4.42 × 10^−4^	2.583	6.27 × 10^−3^	0.00 × 10^−0^
Acetoacetate	0.596	1.01 × 10^−4^	1.662	7.20 × 10^−3^	4.23 × 10^−3^
2-Oxobutanoate	0.593	1.01 × 10^−4^	1.662	3.33 × 10^−3^	4.23 × 10^−3^
GTP	1.920	2.67 × 10^−2^	1.653	4.27 × 10^−3^	7.70 × 10^−3^
N-Carbamoyl-L-Aspartate	0.822	1.50 × 10^−3^	1.604	6.80 × 10^−3^	5.06 × 10^−3^
D-Gluconate	0.725	5.43 × 10^−3^	1.239	2.27 × 10^−3^	1.52 × 10^−2^
Homoserine	0.598	4.21 × 10^−3^	1.581	5.47 × 10^−3^	5.68 × 10^−3^
Acetyl-CoA	0.044	1.07 × 10^−4^	4.158	3.93 × 10^−3^	0.00 × 10^−0^
N-Acetyl-L-Aspartic Acid	2.249	9.87 × 10^−6^	2.106	4.67 × 10^−3^	0.00 × 10^−0^
Adenylosuccinate	0.534	6.10 × 10^−3^	1.746	5.77 × 10^−3^	5.06 × 10^−3^
GDP	2.054	3.16 × 10^−2^	1.693	4.33 × 10^−3^	7.70 × 10^−3^
5-Phosphoribosyl-1-Pyrophosphate	1.758	9.06 × 10^−4^	1.691	2.27 × 10^−3^	4.49× 10^−3^
Cytidine	0.160	2.42 × 10^−4^	3.065	7.13 × 10^−3^	0.00 × 10^−0^
S-Ribosyl-L-Homocysteine	1.416	1.58 × 10^−2^	1.261	6.67 × 10^−3^	1.60 × 10^−2^
Acetylcarnitine DL	0.666	1.50 × 10^−3^	1.428	2.33 × 10^−3^	7.36 × 10^−3^
N-Acetyl-Glutamine	1.608	1.48 × 10^−2^	1.462	1.73 × 10^−3^	1.13 × 10^−2^
Deoxyguanosine	0.563	3.64 × 10^−3^	1.658	2.47 × 10^−3^	5.06 × 10^−3^
Betaine Aldehyde	0.702	4.21 × 10^−3^	1.312	4.80 × 10^−3^	1.21 × 10^−2^
1,3-Diphopshateglycerate	1.843	2.56 × 10^−2^	1.615	4.13 × 10^−3^	8.98 × 10^−3^
Homocysteine	1.377	1.74 × 10^−3^	1.268	5.73 × 10^−3^	1.21 × 10^−2^
dAMP	1.373	2.66 × 10^−3^	1.238	5.40 × 10^−3^	1.41 × 10^−2^
D-Glucono-1,5-Lactone-6-Phosphate	0.700	1.18 × 10^−2^	1.270	1.80 × 10^−3^	1.55 × 10^−2^
Homocysteic Acid	0.617	2.17 × 10^−2^	1.418	1.47 × 10^−3^	1.32 × 10^−2^
Cystine	0.215	1.65 × 10^−2^	2.371	4.80 × 10^−3^	3.85 × 10^−3^
4-Aminobutyrate	0.741	1.86 × 10^−3^	1.218	7.87 × 10^−3^	1.41 × 10^−2^
Putrescine	0.528	2.26 × 10^−3^	1.760	7.60 × 10^−3^	4.49 × 10^−3^
Ornithine	1.405	1.04 × 10^−5^	1.360	7.77 × 10^−3^	5.06 × 10^−3^
Coenzyme A	4.070	1.65 × 10^−2^	2.276	4.13 × 10^−3^	4.23 × 10^−3^
2,3-Diphosphoglyceric Acid	1.926	1.21 × 10^−2^	1.698	4.80 × 10^−3^	5.58 × 10^−3^
Hypoxanthine	0.251	4.76 × 10^−7^	2.770	7.53 × 10^−3^	0.00 × 10^−0^
Citrate	1.399	1.56 × 10^−4^	1.329	9.27 × 10^−3^	7.36 × 10^−3^
Allantoate	0.625	2.42 × 10^−4^	1.567	1.00 × 10^−3^	4.74 × 10^−3^
1-Methyladenosine	0.615	1.69 × 10^−3^	1.543	2.33 × 10^−3^	5.06 × 10^−3^

**Table 4:** Discriminant metabolites identified after treatment with Leflunomide. Test statistics calculated for significantly altered metabolites overlapped across the univariate Student’s *t*-test, SAM, PLS-DA, and RF analysis.

Sample Name	Fold Change	Univariate FDR	VIP Score (Comp 1)	Mean Decrease Accuracy (MDA)	SAM FDR
Citrate-Isocitrate	0.654	2.70 × 10^−5^	1.160	3.97 × 10^−3^	7.57 × 10^−3^
CDP	1.771	1.23 × 10^−3^	1.304	5.13 × 10^−3^	7.32 × 10^−3^
Carbamoyl Phosphate	2.619	5.46 × 10^−5^	1.742	7.00 × 10^−3^	1.11 × 10^−3^
Fumarate	2.787	2.11 × 10^−8^	1.846	2.51 × 10^−3^	2.55 × 10^−4^
Aminoimidazole Carboxamide Ribonucleotide	0.526	4.79 × 10^−3^	1.358	1.06 × 10^−2^	7.41 × 10^−3^
Choline	0.534	1.20 × 10^−2^	1.277	2.00 × 10^−3^	8.62 × 10^−3^
Orotate	15.739	5.83 × 10^−3^	2.569	8.00 × 10^−4^	7.27 × 10^−4^
Thiamine Pyrophosphate	1.781	2.86 × 10^−3^	1.267	3.43 × 10^−3^	7.57 × 10^−3^
Acetoacetate	0.587	2.28 × 10^−2^	1.150	2.27 × 10^−3^	1.55 × 10^−2^
Phosphorylcholine	0.373	1.28 × 10^−9^	1.814	1.15 × 10^−2^	2.55 × 10^−4^
Isocitrate	0.477	4.99 × 10^−3^	1.416	2.13 × 10^−3^	7.32 × 10^−3^
Deoxyadenosine	0.288	1.86 × 10^−2^	1.907	9.00 × 10^−4^	5.73 × 10^−3^
1-Methyladenosine	1.883	3.60 × 10^−3^	1.360	6.67 × 10^−3^	7.32 × 10^−3^
2-Aminooctanoic Acid	1.812	4.99 × 10^−3^	1.267	5.74 × 10^−3^	7.64 × 10^−3^
D-Gluconate	1.999	3.90 × 10^−6^	1.507	5.73 × 10^−3^	3.40 × 10^−3^
2-Keto-Isovalerate	2.868	1.39 × 10^−8^	1.873	5.74 × 10^−3^	2.55 × 10^−4^
Acetyl-CoA	4.344	1.67 × 10^−2^	2.061	8.00 × 10^−4^	4.20 × 10^−3^
N-Carbamoyl-L-Aspartate	51.968	2.86 × 10^−10^	3.627	7.40 × 10^−3^	0.00 × 10^−0^
Cellobiose	0.134	4.99 × 10^−3^	2.296	5.47 × 10^−3^	1.02 × 10^−3^
O8P-O1P	1.511	7.57 × 10^−4^	1.119	8.53 × 10^−3^	9.11 × 10^−3^
Thiamine-Phosphate	1.822	4.87 × 10^−2^	1.147	5.00 × 10^−4^	1.88 × 10^−2^
Creatine	1.814	3.86 × 10^−6^	1.394	4.33 × 10^−3^	5.44 × 10^−3^
CDP-Ethanolamine	7.035	1.45 × 10^−4^	2.473	5.00 × 10^−3^	0.00 × 10^−0^
Acetylcarnitine DL	1.841	3.38 × 10^−3^	1.295	4.13 × 10^−3^	7.57 × 10^−3^
Aconitate	0.542	2.70 × 10^−5^	1.399	9.03 × 10^−3^	5.73 × 10^−3^
Shikimate	0.367	1.81 × 10^−4^	1.781	5.27 × 10^−3^	1.20 × 10^−3^
Anthranilate	1.485	2.12 × 10^−2^	1.014	2.33 × 10^−3^	2.12 × 10^−2^
Uridine	3.660	9.62 × 10^−5^	2.051	1.14 × 10^−3^	7.27 × 10^−4^
2-Isopropylmalic Acid	81.792	1.87 × 10^−11^	3.844	9.53 × 10^−3^	0.00 × 10^−0^
CMP	3.127	3.90 × 10^−6^	1.910	4.07 × 10^−3^	4.24 × 10^−4^
CDP-Choline	8.569	5.71 × 10^−4^	2.438	3.60 × 10^−3^	2.55 × 10^−4^
Deoxyguanosine	0.507	2.09 × 10^−3^	1.402	2.60 × 10^−3^	7.32 × 10^−3^
Citraconic Acid	0.639	1.81 × 10^−4^	1.173	4.73 × 10^−3^	7.57 × 10^−3^
N-acetyl-glucosamine	1.556	8.59 × 10^−3^	1.109	5.47 × 10^−3^	1.40 × 10^−2^
Glycerophosphocholine	1.606	3.48 × 10^−5^	1.225	4.00 × 10^−3^	7.32 × 10^−3^
2-Oxo-4-Methylthiobutanoate	0.462	4.38 × 10^−2^	1.405	1.40 × 10^−3^	9.41 × 10^−3^
Histidinol	1.546	2.44 × 10^−2^	1.021	3.20 × 10^−3^	2.12 × 10^−2^
4-Aminobutyrate	1.948	4.26 × 10^−4^	1.417	4.60 × 10^−3^	6.11 × 10^−3^
Dihydroorotate	7.471	3.97 × 10^−7^	2.569	7.60 × 10^−3^	0.00 × 10^−0^
UDP	1.854	4.22 × 10^−3^	1.315	6.60 × 10^−3^	7.57 × 10^−3^
Itaconic Acid	0.688	8.04 × 10^−4^	1.050	6.07 × 10^−3^	1.23 × 10^−2^
Maleic Acid	2.836	1.41 × 10^−7^	1.860	4.80 × 10^−3^	4.24 × 10^−4^
dCDP	1.194	5.78 × 10^−3^	1.310	4.67 × 10^−3^	7.64 × 10^−3^
N-Acetyl-Glucosamine-1-Phosphate	2.279	4.99 × 10^−3^	1.567	8.00 × 10^−4^	6.46 × 10^−3^
Aspartate	3.759	4.98 × 10^−8^	2.087	1.08 × 10^−2^	0.00 × 10^−0^
Allantoate	158.360	3.66 × 10^−12^	4.120	8.40 × 10^−3^	0.00 × 10^−0^
Guanosine	2.804	2.39 × 10^−3^	1.768	5.00 × 10^−3^	3.89 × 10^−3^

**Table 5:** Significantly altered pathways from overlapped discriminant metabolite sets.

Pathway Name	Percent Affected	Differentiated Metabolites	FDR	Impact
	Tet-On Mfn2 (Direct Fusion)			
Beta-Alanine Metabolism	9.5%	2/21	6.74 × 10^−7^	0.048
Aminoacyl-tRNA Biosynthesis	16.7%	8/48	6.74 × 10^−7^	0.310
Alanine, Aspartate, and Glutamate Metabolism *	28.6%	8/28	7.36 × 10^−6^	0.313
Arginine Biosynthesis *	42.9%	6/14	7.36 × 10^−6^	0.563
Glutathione Metabolism *	14.3%	4/28	7.36 × 10^−6^	0.162
Pantothenate and CoA Biosynthesis	15.8%	3/19	7.85 × 10^−6^	0.111
Glycine, Serine, and Threonine Metabolism	17.7%	6/34	2.00 × 10^−5^	0.262
Nicotinate and Nicotinamide Metabolism	13.3%	2/15	2.83 × 10^−5^	0.190
Pyrimidine Metabolism *	23.1%	9/39	4.87 × 10^−5^	0.220
Arginine and Proline Metabolism	13.2%	5/38	4.88 × 10^−5^	0.300
Cysteine and Methionine Metabolism *	15.2%	5/33	5.28 × 10^−5^	0.152
Amino Sugar and Nucleotide Sugar Metabolism	5.4%	2/37	1.85 × 10^−4^	0.087
Pentose Phosphate Pathway *	13.6%	3/22	2.16 × 10^−4^	0.103
Glycolysis/Gluconeogenesis *	3.9%	1/26	2.64 × 10^−4^	0.029
Purine Metabolism *	10.6%	7/66	4.70 × 10^−4^	0.136
	sgDrp1 (Indirect Fusion)			
Arginine Biosynthesis *	14.3%	2/14	2.05 × 10^−7^	0.1875
Alanine, Aspartate, and Glutamate Metabolism *	21.4%	6/28	2.32 × 10^−7^	0.28125
Fatty Acid Degradation	5.1%	2/39	2.32 × 10^−7^	0.16327
Arginine and Proline Metabolism	15.8%	6/38	2.66 × 10^−7^	0.25
Glutathione Metabolism *	17.9%	5/28	4.20 × 10^−7^	0.27028
Glycolysis/Gluconeogenesis *	11.5%	3/26	1.56 × 10^−6^	0.11428
Pyrimidine Metabolism *	7.7%	3/39	1.82 × 10^−6^	0.0339
Nitrogen Metabolism	16.7%	1/6	2.85 × 10^−6^	0.25
Pentose Phosphate Pathway *	18.2%	4/22	3.06 × 10^−6^	0.10344
Glyoxylate and Dicarboxylate Metabolism	9.4%	3/32	3.06 × 10^−6^	0.11538
Purine Metabolism *	15.2%	10/66	3.92 × 10^−6^	0.2159
Butanoate Metabolism	26.7%	4/15	7.42 × 10^−6^	0.33334
Citrate Cycle (TCA cycle)	10%	2/20	7.42 × 10^−6^	0.10345
Propanoate Metabolism	8.7%	2/23	7.69 × 10^−6^	0.11538
Cysteine and Methionine Metabolism *	12.1%	4/33	3.69 × 10^−5^	0.15151
	Leflunomide (Pharmacologic)			
Alanine, Aspartate, and Glutamate Metabolism *	25%	7/28	3.28 × 10^−9^	0.313
Pantothenate and CoA Biosynthesis	10.5%	2/19	2.69 × 10^−8^	0.056
Pyrimidine Metabolism *	25.6%	10/39	2.69 × 10^−8^	0.288
Purine Metabolism *	9.1%	6/66	2.25 × 10^−7^	0.102
Citrate Cycle (TCA cycle)	30%	6/20	5.03 × 10^−7^	0.276
Valine, Leucine, and Isoleucine Biosynthesis	12.5%	1/8	5.03 × 10^−7^	0.250
Aminoacyl-tRNA Biosynthesis	2.1%	1/48	5.03 × 10^−7^	0.034
Glyoxylate and Dicarboxylate Metabolism	12.5%	4/32	7.65 × 10^−7^	0.154
Nicotinate and Nicotinamide Metabolism	13.3%	2/15	7.65 × 10^−7^	0.190
Arginine Biosynthesis *	21.4%	3/14	8.20 × 10^−7^	0.125
Glycine, Serine, and Threonine Metabolism	5.9%	2/34	4.87 × 10^−5^	0.024
Butanoate Metabolism	26.7%	4/15	8.19 × 10^−5^	0.267
Valine, Leucine, and Isoleucine Degradation	10%	4/40	3.43 × 10^−4^	0.132
Cysteine and Methionine Metabolism *	3.0%	1/33	1.55 × 10^−3^	0.030
Beta-Alanine Metabolism	9.5%	2/21	2.94 × 10^−3^	0.048
Pentose Phosphate Pathway *	9.1%	2/22	1.10 × 10^−2^	0.069
Glutathione Metabolism *	3.6%	1/28	4.23 × 10^−2^	0.027
Glycolysis/Gluconeogenesis *	7.7%	2/26	4.37 × 10^−2^	0.057

* Pathways common amongst Tet-On Mfn2, sgDrp1, and Lef treatment.

## Data Availability

Raw data files can be found in the Supplementary Materials.

## Supplementary Materials

The following are available online at https://www.mdpi.com/article/10.3390/metabo11090627/s1, Figure S1: Western blot analysis confirms direct induction of mitochondrial fusion occurs independent of Drp1 expression. (**A**) tetracycline-inducible Mfn2, (**B**) Drp1 knockout, and (**C**) leflunomide treated KPC cells were probed for Mfn2, Drp1, and Vinculin as a loading control, Figure S2: Pathway analysis generated from discriminant metabolites identified by univariate Student’s *t*-test, Figure S3: Pathway analysis generated from discriminant metabolites identified by SAM, Figure S4: Pathway analysis generated from discriminant metabolites identified by PLS-DA VIP, Figure S5: Pathway analysis generated from discriminant metabolites identified by RF classification, Figure S6: Significantly altered metabolic pathways in fusion induced KPC cells interconnected, Figure S7: Full uncut western blots, Table S1: Total pathway analysis after inducing mitochondrial fusion through Tet-On Mfn2, sgDrp1, and Lef, Table S2: (**A**–**C**) Discriminant Metabolites *t*-test, Table S3: (**A**–**C**) Discriminant Metabolites SAM, Table S4: (**A**–**C**) Discriminant Metabolites PLS-DA VIP Score, Table S5: (**A**–**C**) Discriminant Metabolites RF, Table S6: (**A**–**C**) Pathway Analysis *t*-test, Table S7: (**A**–**C**) Pathway Analysis SAM, Table S8: (**A**–**C**) Pathway Analysis VIP, Table S9: (**A**–**C**) Pathway Analysis RF, Table S10: (**A**–**C**) Pathway Analysis Overlapped.
