# Supplementary material for: Comparative Untargeted Metabolomic Profiling of Induced Mitochondrial Fusion in Pancreatic Cancer
Source: Metabolites. 2021 Sep 15;11(9):627. doi: 10.3390/metabo11090627 (PMC8470144; doi:10.3390/metabo11090627)

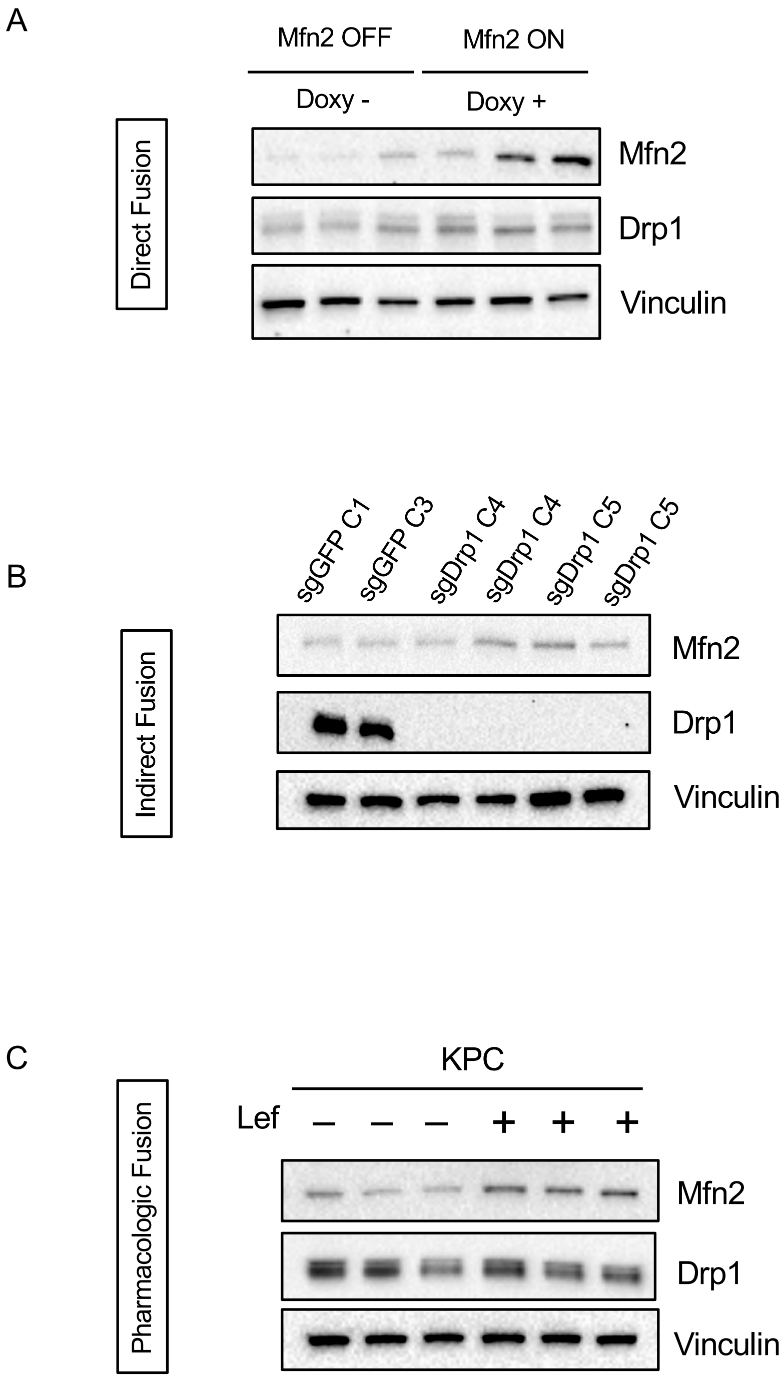

Figure S1. Western blot analysis confirms direct induction of mitochondrial fusion occurs independent of Drp1 expression. (**A**) tetracycline-inducible Mfn2, (**B**) Drp1 knockout, and (**C**) leflunomide treated KPC cells were probed for Mfn2, Drp1, and Vinculin as a loading control.

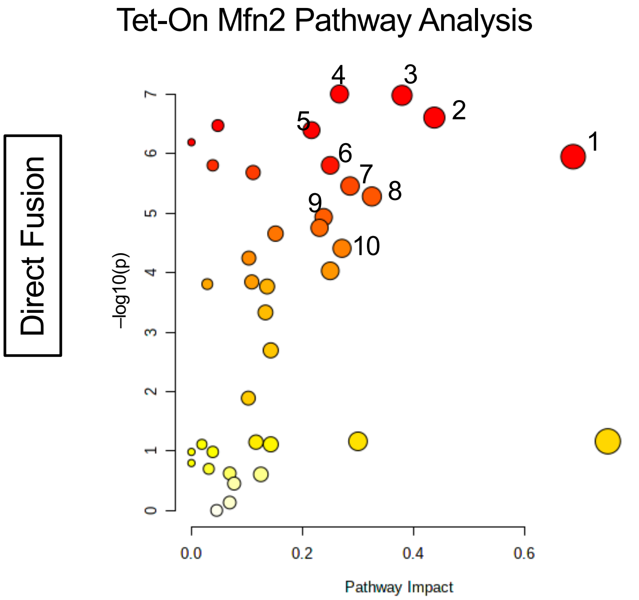

1. Arginine Biosynthesis
2. Alanine, Aspartate, and Glutamate Metabolism
3. Aminoacyl-tRNA Biosynthesis
4. Histidine Metabolism
5. Glutathione Metabolism
6. D-Glutamine and D-Glutamate Metabolism
7. Glycine, Serine, and Threonine Metabolism
8. Arginine and Proline Metabolism
9. Nicotinate and Nicotinamide Metabolism
10. Pyrimidine Metabolism

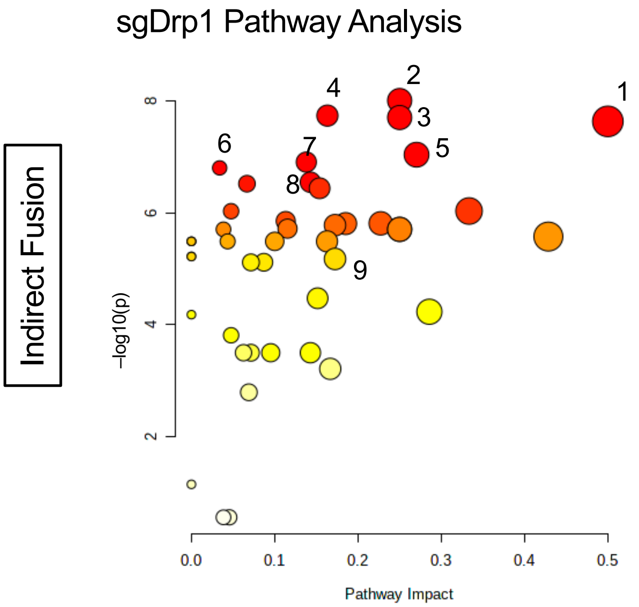

1. Alanine, Aspartate, and Glutamate Metabolism
2. Arginine and Proline Metabolism
3. Arginine Biosynthesis
4. Fatty Acid Degradation
5. Glutathione Metabolism
6. Pyrimidine Metabolism
7. Aminoacyl-tRNA Biosynthesis
8. Glycolysis/Gluconeogenesis
9. Pentose Phosphate Pathway

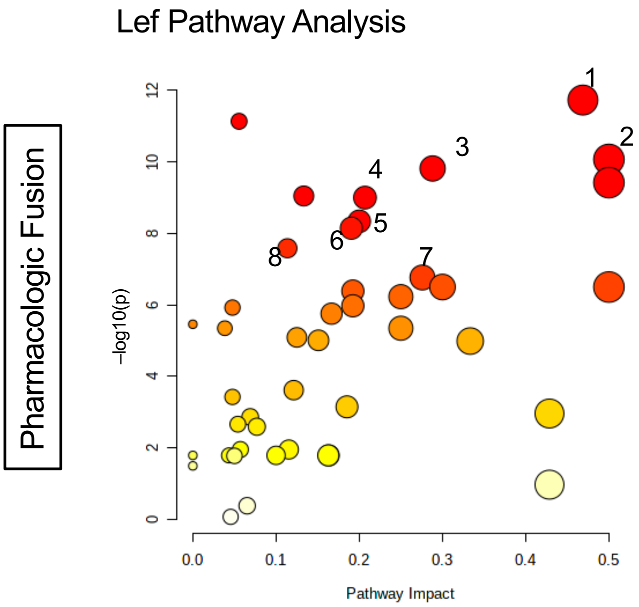

1. Alanine, Aspartate, and Glutamate Metabolism
2. Arginine Biosynthesis
3. Pyrimidine Metabolism
4. Aminoacyl t-RNA Metabolism
5. Arginine and Proline Metabolism
6. Nicotinate and Nicotinamide Metabolism
7. TCA Cycle
8. Purine Metabolism

Figure S2. Pathway analysis generated from discriminant metabolites identified by univariate Student's *t*-test.

Tet-On Mfn2 Pathway Analysis

Direct Fusion

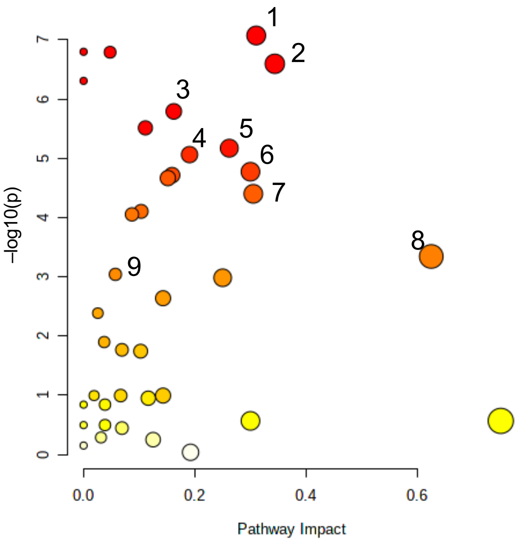

1. Alanine, Aspartate, and Glutamate Metabolism
2. Arginine Biosynthesis
3. Pyrimidine Metabolism
4. Aminoacyl t-RNA Metabolism
5. Arginine and Proline Metabolism
6. Nicotinate and Nicotinamide Metabolism
7. TCA Cycle
8. Purine Metabolism

sgDrp1 Pathway Analysis

Indirect Fusion

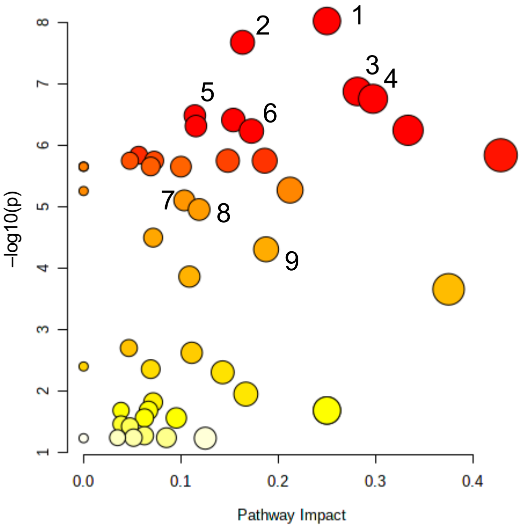

1. Arginine and Proline Metabolism
2. Fatty Acid Degradation
3. Alanine, Aspartate, and Glutamate Metabolism
4. Glutathione Metabolism
5. Glycolysis/Gluconeogenesis
6. TCA Cycle
7. Pentose Phosphate Pathway
8. Pyrimidine Metabolism
9. Arginine Biosynthesis

Lef Pathway Analysis

Pharmacologic Fusion

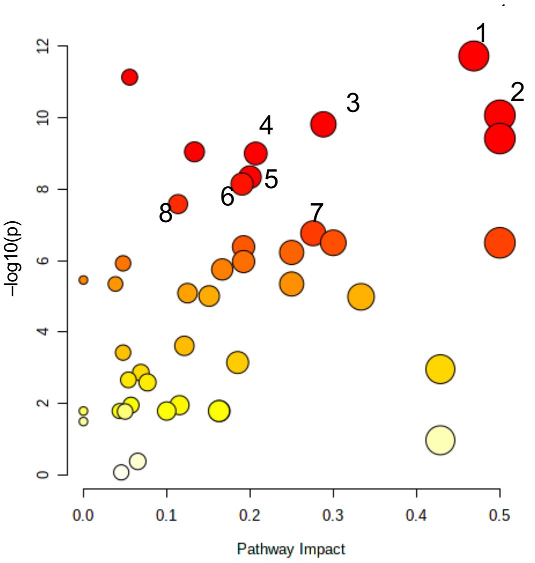

1. Alanine, Aspartate, and Glutamate Metabolism
2. Arginine Biosynthesis
3. Pyrimidine Metabolism
4. Aminoacyl t-RNA Metabolism
5. Arginine and Proline Metabolism
6. Nicotinate and Nicotinamide Metabolism
7. TCA Cycle
8. Purine Metabolism

Figure S3. Pathway analysis generated from discriminant metabolites identified by SAM.

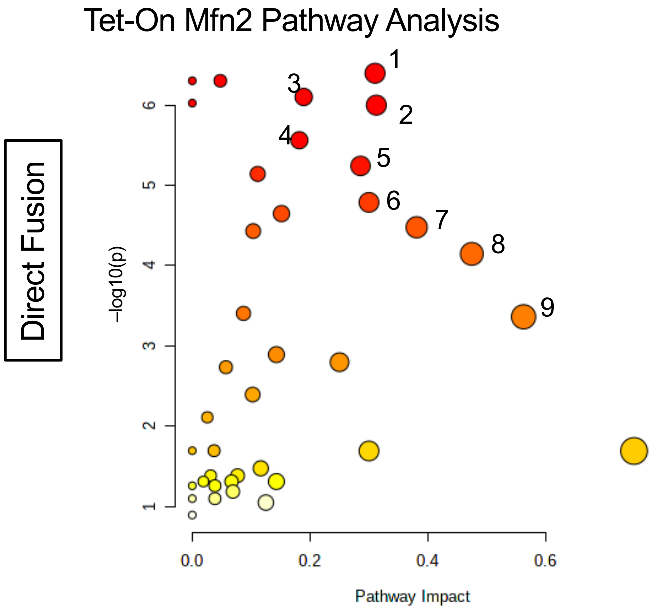

1. Aminoacyl-tRNA Biosynthesis
2. Alanine, Aspartate, and Glutamate Metabolism
3. Glutathione Metabolism
4. Purine Metabolism
5. Glycine, Serine, and Threonine Metabolism
6. Arginine and Proline Metabolism
7. Nicotinate and Nicotinamide Metabolism
8. Pyrimidine Metabolism
9. Arginine Biosynthesis

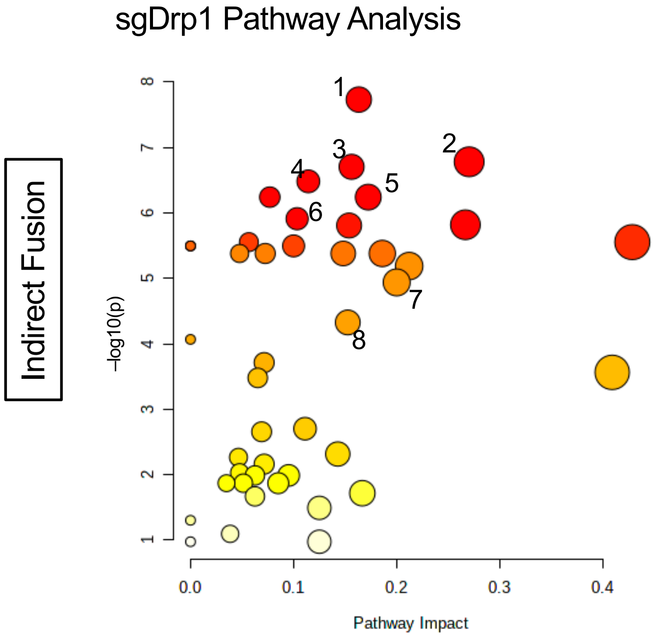

1. Fatty Acid Degradation
2. Glutathione Metabolism
3. Alanine, Aspartate, and Glutamate Metabolism
4. Glycolysis/Gluconeogenesis
5. TCA Cycle
6. Pentose Phosphate Pathway
7. Arginine and Proline Metabolism
8. Pyrimidine Metabolism

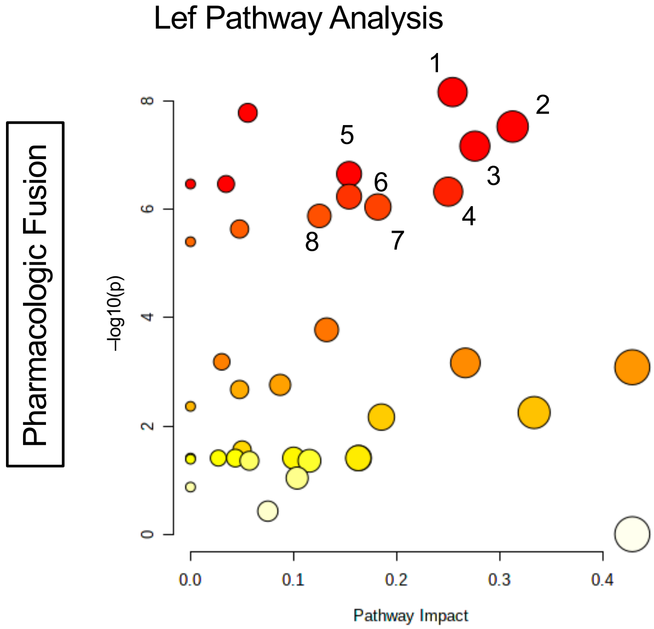

1. Pyrimidine Metabolism
2. Alanine, Aspartate, and Glutamate Metabolism
3. TCA Cycle
4. Valine, Leucine, and Isoleucine Biosynthesis
5. Glycoxylate and Dicarboxylate Metabolism
6. Glycerophospholipid Metabolism
7. Purine Metabolism
8. Arginine Biosynthesis

Figure S4. Pathway analysis generated from discriminant metabolites identified by PLS-DA VIP.

Direct Fusion

Tet-On Mfn2 Pathway Analysis

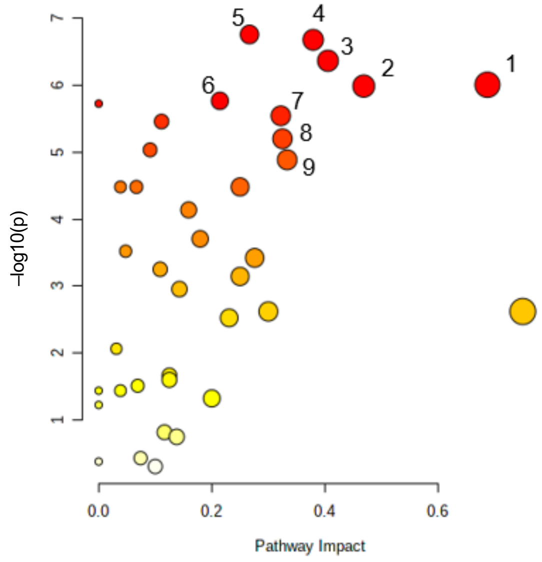

1. Arginine Biosynthesis
2. Alanine, Aspartate, and Glutamate Metabolism
3. Glutathione Metabolism
4. Aminoacyl-tRNA Biosynthesis
5. Histidine Metabolism
6. Glycine, Serine, and Threonine Metabolism
7. Pyrimidine Metabolism
8. Arginine and Proline Metabolism
9. Nicotinate and Nicotinamide Metabolism

Indirect Fusion

sgDrp1 Pathway Analysis

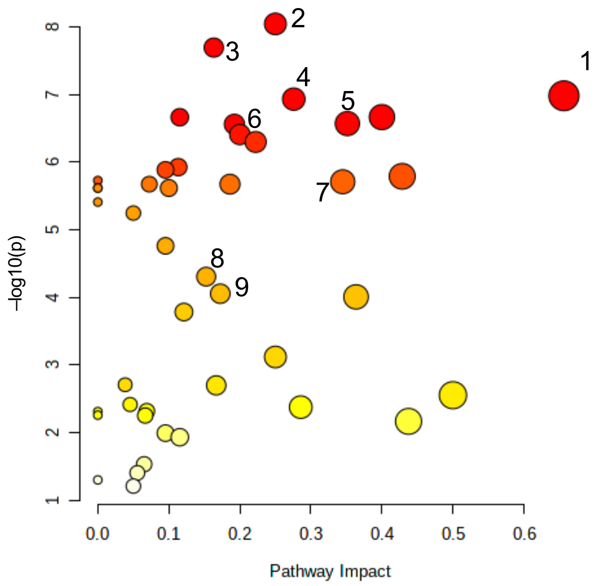

1. Alanine, Aspartate, and Glutamate Metabolism
2. Arginine and Proline Metabolism
3. Arginine Biosynthesis
4. TCA Cycle
5. Glutathione Metabolism
6. Glycolysis/Gluconeogenesis
7. Pentose Phosphate Pathway
8. Pyrimidine Metabolism
9. Aminoacyl-tRNA Biosynthesis

Pharmacologic Fusion

Lef Pathway Analysis

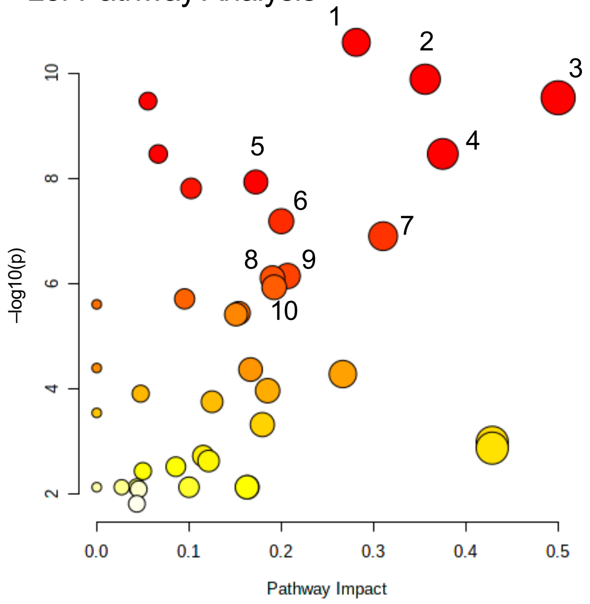

1. Alanine, Aspartate, and Glutamate Metabolism
2. Pyrimidine Metabolism
3. Valine, Leucine, and Isoleucine Biosynthesis
4. Arginine Biosynthesis
5. Aminoacyl t-RNA Metabolism
6. Arginine and Proline Metabolism
7. TCA Cycle
8. Pentose Phosphate Pathway
9. Nicotinate and Nicotinamide Metabolism
10. Glycerophospholipid Metabolism

Figure S5. Pathway analysis generated from discriminant metabolites identified by RF classification.

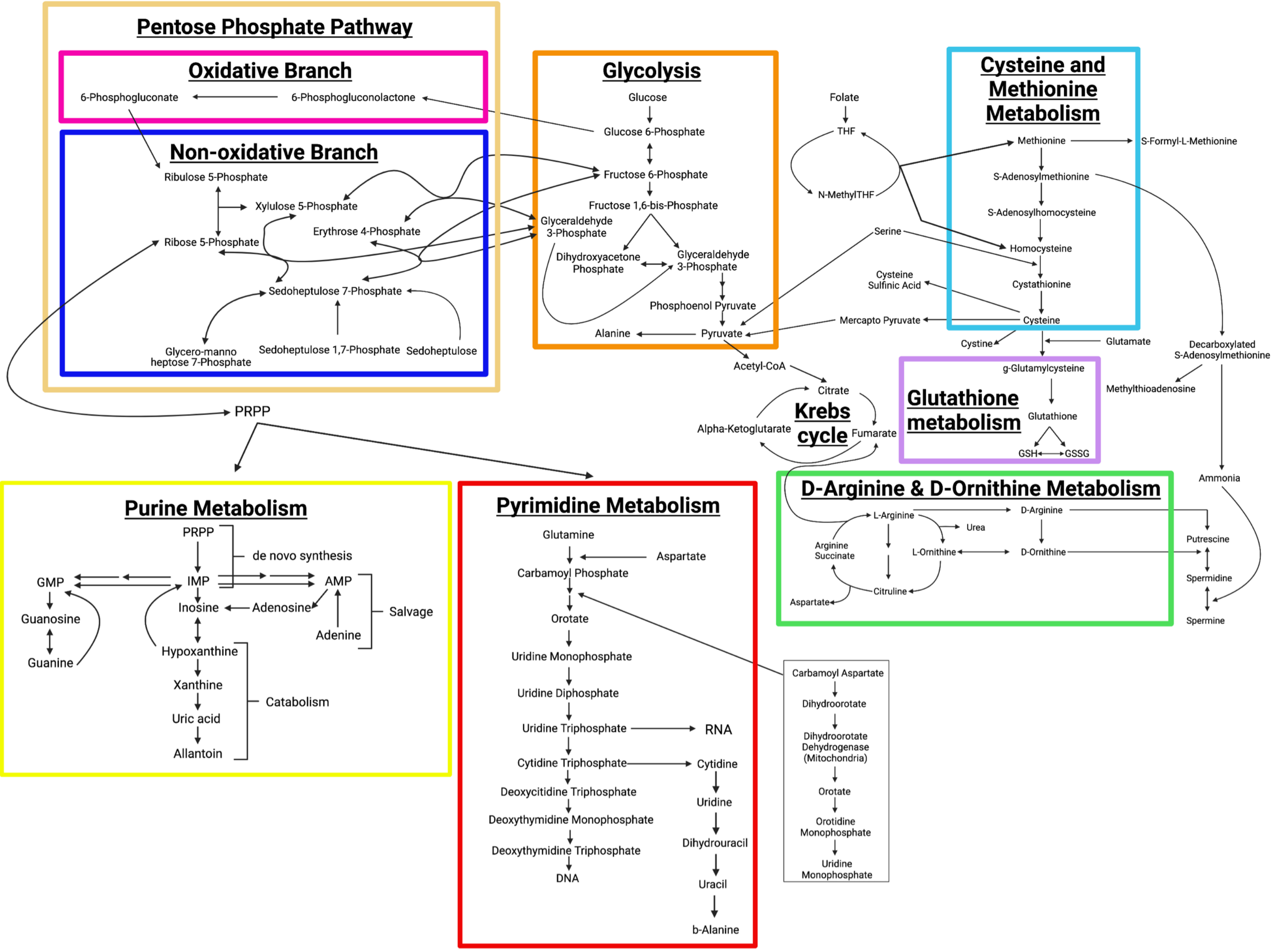

Figure S6. Significantly altered metabolic pathways in fusion induced KPC cells interconnected. Top 8 altered metabolic pathways identified from our overlapped discriminant metabolite set in fused KPC cells are mapped to show their interdependent relationships among each other.

Figure S7. Full Uncut Western Blots

From Figure S6A

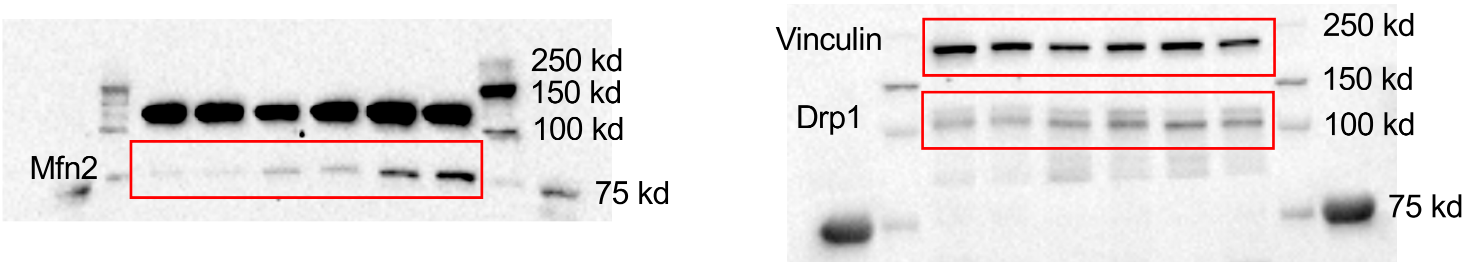

From Figure S6B

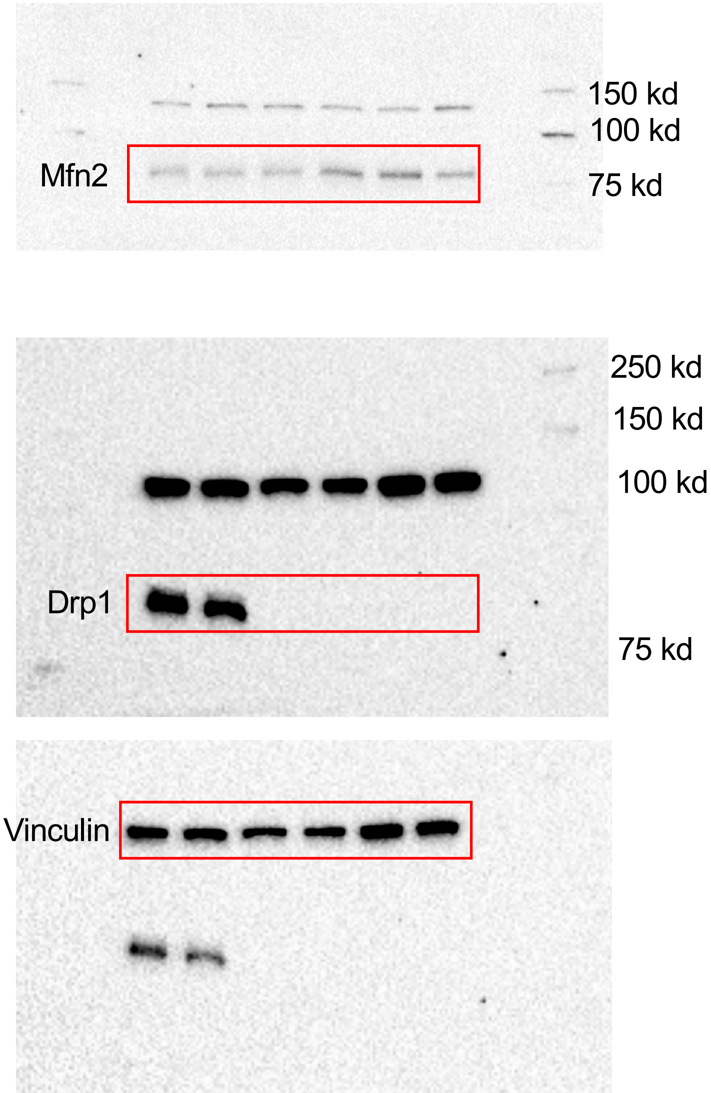

From Figure S6C

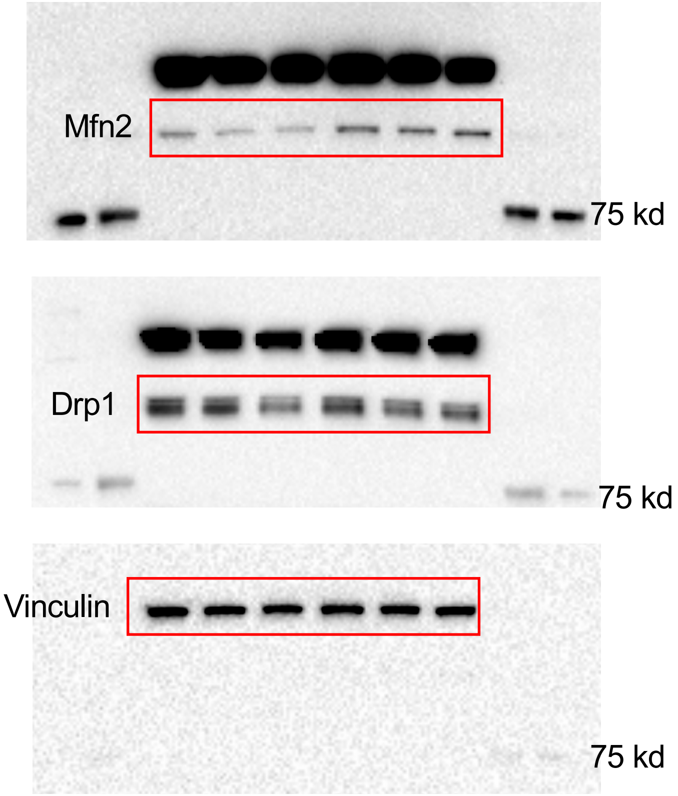

Supplement: Supplementary file 1 [file metabolites-11-00627-s001.zip › Supplementary Figures and Tables for Revisions Proof/Supplementary Figures S1-6.pdf]
